# Comparative Immunopathology of *Cetacean morbillivirus* Infection in Free-Ranging Dolphins From Western Mediterranean, Northeast-Central, and Southwestern Atlantic

**DOI:** 10.3389/fimmu.2019.00485

**Published:** 2019-03-18

**Authors:** Josué Díaz-Delgado, Kátia R. Groch, Rodrigo Ressio, Isis P. J. Riskallah, Eva Sierra, Simona Sacchini, Óscar Quesada-Canales, Manuel Arbelo, Antonio Fernández, Elitieri Santos-Neto, Joana Ikeda, Rafael Ramos de Carvalho, Alexandre de Freitas Azevedo, Jose Lailson-Brito, Leonardo Flach, Cristina T. Kanamura, Natália C. C. A. Fernandes, Bruno Cogliati, Cinzia Centelleghe, Sandro Mazzariol, Ludovica Di Renzo, Gabriella Di Francesco, Giovanni Di Guardo, José Luiz Catão-Dias

**Affiliations:** ^1^Laboratory of Wildlife Comparative Pathology, Department of Pathology, School of Veterinary Medicine and Animal Science, University of São Paulo, São Paulo, Brazil; ^2^Pathology Center, Adolfo Lutz Institute, São Paulo, Brazil; ^3^Laboratory of Morphologic and Molecular Pathology, Department of Pathology, School of Veterinary Medicine and Animal Science, University of São Paulo, São Paulo, Brazil; ^4^School of Veterinary Medicine, Institute for Animal Health and Food Safety, University of Las Palmas of Gran Canaria, Arucas, Spain; ^5^Laboratory of Aquatic Mammals and Bioindicators: Profa Izabel M. G. do N. Gurgel' (MAQUA), Faculty of Oceanography, Rio de Janeiro State University, Rio de Janeiro, Brazil; ^6^Instituto Boto Cinza, Mangaratiba, Rio de Janeiro, Brazil; ^7^Department of Comparative Biomedicine and Food Hygiene (BCA), University of Padova, Legnaro, Italy; ^8^Istituto Zooprofilattico Sperimentale dell'Abruzzo e del Molise “G. Caporale”, Teramo, Italy; ^9^Faculty of Veterinary Medicine, University of Teramo, Teramo, Italy

**Keywords:** *Cetacean morbillivirus*, immunopathogenesis, neuroimmunopathology, lymphocytes, histiocytes, apoptosis, cytokines

## Abstract

*Cetacean morbillivirus* (CeMV; *Paramyxoviridae*) causes epizootic and interepizootic fatalities in odontocetes and mysticetes worldwide. Studies suggest there is different species-specific susceptibility to CeMV infection, with striped dolphins (*Stenella coeruleoalba*), bottlenose dolphins (*Tursiops truncatus*), and Guiana dolphins (*Sotalia guianensis*) ranking among the most susceptible cetacean hosts. The pathogenesis of CeMV infection is not fully resolved. Since no previous studies have evaluated the organ-specific immunopathogenetic features of CeMV infection in tissues from infected dolphins, this study was aimed at characterizing and comparing immunophenotypic profiles of local immune responses in lymphoid organs (lymph nodes, spleen), lung and CNS in CeMV-molecularly (RT-PCR)-positive cetaceans from Western Mediterranean, Northeast-Central, and Southwestern Atlantic. Immunohistochemical (IHC) analyses targeted molecules of immunologic interest: caspase 3, CD3, CD20, CD57, CD68, FoxP3, MHCII, Iba1, IFNγ, IgG, IL4, IL10, lysozyme, TGFβ, and PAX5. We detected consistent CeMV-associated inflammatory response patterns. Within CNS, inflammation was dominated by CD3^+^ (T cells), and CD20^+^ and PAX5^+^ (B cells) lymphocytes, accompanied by fewer Iba1^+^, CD68^+^, and lysozyme^+^ histiocytes, mainly in striped dolphins and bottlenose dolphins. Multicentric lymphoid depletion was characterized by reduced numbers of T cells and B cells, more pronounced in Guiana dolphins. Striped dolphins and bottlenose dolphins often had hyperplastic (regenerative) phenomena involving the aforementioned cell populations, particularly chronically infected animals. In the lung, there was mild to moderate increase in T cells, B cells, and histiocytes. Additionally, there was a generalized increased expression of caspase 3 in lymphoid, lung, and CNS tissues. Apoptosis, therefore, is believed to play a major role in generalized lymphoid depletion and likely overt immunosuppression during CeMV infection. No differences were detected regarding cytokine immunoreactivity in lymph nodes, spleen, and lung from infected and non-infected dolphins by semiquantitative analysis; however, there was striking immunoreactivity for IFNγ in the CNS of infected dolphins. These novel results set the basis for tissue-specific immunophenotypic responses during CeMV infection in three highly susceptible delphinid species. They also suggest a complex interplay between viral and host's immune factors, thereby contributing to gain valuable insights into similarities, and differences of CeMV infection's immunopathogenesis in relation to body tissues, CeMV strains, and cetacean hosts.

## Introduction

*Cetacean morbillivirus* (CeMV; genus *Morbillivirus*, family *Paramyxoviridae*) has caused multiple outbreaks of lethal disease in odontocetes and mysticetes worldwide. Interepizootic or endemic morbidity and mortality is also recorded (1). There are three well characterized CeMV strains (porpoise MV, dolphin [D]-MV, and pilot whale MV) mainly in northern hemisphere, and three novel strains, one of them detected in Brazil, i.e., Guiana dolphin (GD)-MV, which is also considered the first reported example of CeMV infection among cetaceans from South America (2, 3). Studies suggest there is different species-specific susceptibility to CeMV infection with bottlenose dolphins (*Tursiops truncatus*), striped dolphins (*Stenella coeruleoalba*), and Guiana dolphins (*Sotalia guianensis*) ranking among the most susceptible cetacean hosts, with fatal epizootics (1, 3, 4). CeMV may cause severe lymphoid, respiratory, and neurologic disease in susceptible species, leading to stranding and death. Four major presentations of CeMV-associated pathology (CeMV-AP) are currently recognized, which bear resemblance to the pathologic features of measles virus (MeV) and canine distemper virus (CDV) infections, the major morbilliviral diseases in humans and dogs, respectively (5, 6).

The pathogenesis of infections by terrestrial morbilliviruses (TMVs) involve initial replication in lymphoid tissues, followed by viral dissemination in infected lymphocytes through the lymphatics and blood stream (“leukocyte trafficking”), and eventual spread to epithelial and nervous cells (6, 7). Immunohistochemical (IHC) studies on naturally occurring CeMV infections in cetaceans support the above pathogenesis, with a predominant aerogenous transmission (1). Several studies have focused on the immunophenotypic characterization of local inflammatory responses (LIRs) in TMV infections, especially in measles (in both humans and non-human primates) and distemper (in canids and susceptible non-canid carnivore species), with major emphasis on lymphoid tissues, lung, and central nervous system (CNS) (8, 9). In measles and distemper, various lymphocytic, histiocytic, and cytokine patterns have been shown to vary depending on disease chronology and other factors. Furthermore, cytokine imbalance in Th1 and Th2 immune responses (early and advanced disease stages, respectively) plays a major role in disease susceptibility and progression in MeV- and CDV-infected individuals (5, 8, 9). Nevertheless, there are no published studies focused on the pathogenetic evolution of CeMV infection in Th1-dominant vs. Th2-dominant cetacean hosts (10). Immunophenotypic studies on LIR during CeMV infection are also lacking, except for a previous study focused on peripheral blood leukocytes (PBLs) in a set of bottlenose dolphins with subclinical infection (11). In order to partially fill in this knowledge gap, the present study was aimed at characterizing and comparing the immunophenotypic profiles of CeMV-associated LIR in lymphoid, lung and CNS tissues of infected cetaceans from Western Mediterranean (Italy), Northeast-Central (Canary Islands), and Southwestern Atlantic (Brazil).

## Material and Methods

### Data and Sample Collection

The marine mammal databases and tissue banks of collaborative research institutions, namely the “Laboratory of Wildlife Comparative Pathology—LAPCOM” (São Paulo, Brazil), the “Laboratory of Aquatic Mammals and Bioindicators Profa. Izabel M. G. do N. Gurgel—MAQUA” (Rio de Janeiro, Brazil), the “Institute for Animal Health and Food Safety—IUSA” (Canary Islands, Spain), the “Department of Comparative Biomedicine and Food Science of the Faculty of Veterinary Medicine of the University of Padua (Legnaro, Italy),” the “Laboratories of Histopathology and Immunohistochemistry of Istituto Zooprofilattico Sperimentale dell'Abruzzo e Molise G. Caporale,” and the “Faculty of Veterinary Medicine of the University of Teramo (Teramo, Italy),” were queried based upon the following criteria: “*Sotalia guianensis*,” “*Stenella coeruleoalba*,” “*Tursiops truncatus*,” “CeMV reverse transcription polymerase chain reaction (RT-PCR)-positive” “*Toxoplasma gondii* PCR-negative.” Only individuals in a “fresh” (code 2) *post mortem* preservation *status*, or in a “moderate *post mortem* autolysis” (code 3) condition (12), which could also warrant a sufficient amount of formalin-fixed, paraffin-embedded (FFPE), and frozen tissues for extensive analysis, including immunohistochemical (IHC) and cytokine gene expression investigations (parallel manuscript) on target organs (lymph nodes, spleen, lung, brain), were included. Additionally, tissues from three CeMV-negative dolphins including one striped dolphin (Canary Islands), one bottlenose dolphin (Italy), and one Guiana dolphin (Brazil) that were fresh, in good body condition and died as result of bycatch and/or traumatic interaction(s) and lacked morphological and molecular evidence of any infectious etiology were used as “controls” for IHC comparison purposes. The tissue samples came from complete standard necropsies. Epidemiologic and biologic data, along with necropsy reports, photographic material, and ancillary diagnostic techniques were retrieved and further analyzed. Required permissions for the management of tissues from cetaceans found stranded along the coasts of Brazil, the Canarian archipelago, and Italy were issued by the respective official authorities. All dolphins had spontaneous naturally occurring CeMV infection and no experiments were performed on live animals. Detailed comparative histopathologic investigations and viral IHC results for these animals will be published elsewhere.

### Immunohistochemistry

Selected FFPE tissues including lymph nodes (mediastinal/tracheobronchial, pulmonary, mesenteric, prescapular) and spleen, lung, and CNS (cerebrum, cerebellum, brain stem, spinal cord) were subjected to IHC using the following primary antibodies (pAbs): cleaved caspase 3 (CAS3) [final apoptosis pathway], cluster of differentiation (CD)-3 [T cell], CD20 [B cell], CD57 [natural killer cell], CD68 [histiocyte], Forkhead Box (Fox)-P3 [regulatory T cell], human leukocyte antigen (HLA-DRα; *syn*. major histocompatibility complex II, MHCII) [antigen presenting cell], ionized calcium binding adaptor molecule 1 (Iba1) [histiocyte/microglia], interferon gamma (IFNγ), immunoglobulin (Ig)-G, interleukin (IL)-4, IL10, lysozyme, transforming growth factor beta (TGFβ), and paired box protein (PAX)-5 [B-cell]. Most of this IHC panel was standardized with successful cross-reactivity in cetacean tissues (13). Further details on the IHC protocol are recorded in Table 1. Briefly, serial sections at 3 μm-thick were cut and collected onto coated slides. Antigen retrieval was followed by endogenous peroxidase blocking and nonspecific binding blocking with normal serum of same species where pAbs were raised. PAbs were incubated overnight (18 h, 4C°). Amplification and visualization was achieved by the HiDef DetectionTM HRP Polymer System (Cell Marque, Rocklin, California, USA) followed by diaminobenzidine (DAB D-5637; Sigma, St. Louis, Missouri, USA) chromogen and counterstaining with Harris' haematoxylin. Normal human, mouse and franciscana (*Pontoporia blainvillei*) lymph node, spleen, lung, and brain were used as positive controls (13). Tissue sections in which the pAbs were replaced by non-immune homologous serum served as negative controls.

**Table 1:** Tested antigen, clone, species of origin, clonality, antigen retrieval methods, working dilutions for primary antibodies and amplification and visualization method for immunohistochemistry of selected immune components in formalin-fixed, paraffin-embedded lymphoid tissues of striped dolphins (*Stenella coeruleoalba*), bottlenose dolphins (*Tursiops truncatus*), and Guiana dolphins (*Sotalia guianensis*).

**Antigen**	**Clone**	**Species**	**Clonality**	**Retrieval**	**[Antibody]**	**Amplification/visualization**
Caspase 3^a^	Asp175	Rabbit	Pol	pH6	1:200	PoH/DAB^i^^/^^j^
CD3^b^	A0452	Rabbit	Pol	pH9	1:1000	PoH/DAB^i^
CD20^c^	RB9013P	Rabbit	Pol	pH9	1:100	PoH/DAB^i^
CD57^d^	NKNE1	Mouse	Mon	pH6	1:400	PoH/DAB^i^
CD68^b^	KP1	Mouse	Mon	pH6	1:3000	PoH/DAB^i^
FoxP3^a^	D2W8E	Mouse	Mon	pH9	1:10	PoH/DAB^i^
HLA-DRα^b^	TAL1B5	Mouse	Mon	pH9	1:400	PoH/DAB^i^
Iba-1^e^	NCNP24	Rabbit	Pol	pH9	1:500	PoH/DAB^i^
IFNγ^f^	DBNE1	Mouse	Mon	pH6	1:1000	PoH/DAB^i^
IgG^b^	A0423	Rabbit	Pol	pH9	1:1000	PoH/DAB^i^
IL4^g^	ab9811	Rabbit	Pol	pH9	1:400	PoH/DAB^i^
IL10^g^	ab34843	Rabbit	Pol	pH9	1:400	PoH/DAB^i^
Lysozyme^b^	A0099	Rabbit	Pol	pH6	1:3000	PoH/DAB^i^
TGF-β^g^	213NE4.4	Mouse	Mon	pH9	1:200	PoH/DAB^i^
PAX-5^h^	BC/24	Mouse	Mon	pH9	1:300	PoH/DAB^i^

*CD, cluster of differentiation; FoxP3, Forkhead Box P3; HLA, human leukocyte antigen (synonym major histocompatibility complex); IFN, interferon; Ig, immunoglobulin; IL, interleukin; TGF, transforming growth factor; Pax-5, paired box 5; Mon, monoclonal; Pol, polyclonal; PK, proteinase K; NE: not evaluated; PoH; HiDef Detection™ Polymer System; DAB, Diaminobenzidine. Best antigen retrieval and primary antibody concentrations are indicated in bold. pH6, citrate; pH9, EDTA, Ethylenediamine tetraacetic acid*.

a *Cell signaling (Danvers, MA, USA)*,

b *Dako-Agilent (Santa Clara, CA, USA)*,

c *Thermo (Fremont, CA, USA)*,

d *Novocastra-Leica (Nussloch, Germany)*,

e *Wako Pure Chemical (Richmond, VA, USA)*,

f *Bio-Rad (Hercules, CA, USA)*,

g *Proteintech (Rosemont, IL, USA)*,

h *Biocare (Pacheco, CA, USA)*,

I *Cell Marque (Rocklin, CA, USA)*,

j *DAB, 3,3′-Diaminobenzidine (D-5637; Sigma, St. Louis, Missouri, USA)*.

For immunophenotypic characterization of local inflammatory cell populations, the number of immunopositive cells was semiquantitatively evaluated for each marker in lymphoid (lymph nodes, spleen), lung, and CNS tissues according to following score: –, no; +, <10%; ++, 10–50%; +++, 51–90%, and ++++, >90% immunopositive cells, in 10 high-power (400x) fields. The histo-anatomical compartments analyzed were: lymph nodes (primary and secondary follicles, paracortex, medullary cords and cortical, paracortical, and medullary sinuses; afferent/efferent lymphatics); spleen (follicles, perifollicular zone, periarteriolar lymphoid sheath [PALS], red pulp sinuses, and cords); lung (bronchial/bronchiolar mucosa and submucosa including glands and associated lymphoid tissue [BALT]; alveolar spaces and septa; interstitium; pleura; vasculature including lymphatics); brain (cerebral cortex, cerebellum, brain stem, spinal cord) (13, 14). Additionally, IHC expression intensity was subjectively scored (increasing intensity) as 1, 2, and 3. The results were compared between CeMV-infected and non-infected “control” dolphins.

## Results

Twenty-seven CeMV-positive dolphins, including 13 striped dolphins, 11 Guiana dolphins, and 3 bottlenose dolphins met the inclusion criteria. Guiana dolphins were infected by GD-CeMV (2, 3), while striped dolphins and bottlenose dolphins were infected by DMV (15–18). Epidemiologic and biologic data as well as CeMV-RT-PCR-positive tissues are recorded in Table 2. CeMV-positive animals included: calves (*n* = 2), juveniles (*n* = 11), and adults (*n* = 14). All CeMV-negative dolphins were calves. Detailed gross and microscopic pathologic findings with most probable cause(s) of stranding and/or death for CeMV-positive are published elsewhere and for CeMV-negative dolphins are recorded in Supplemental Table S1.

**Table 2:** Epidemiologic and biologic data of Guiana dolphins (*Sotalia guianensis*), striped dolphins (*Stenella coeruleoalba*), and bottlenose dolphins (*Tursiops truncatus*) included in this study.

**No**	**Species**	**Stranding**	**Coordinates (country)**	**BL (cm)**	**Age**	**Sex**	**NS**	**DC**	**SC**	**CeMV chronicity**
1	*S. guianensis*^a^	09-Nov-2017	23°10′6″S; 44°20′82″W (BR)	177	Ad	Fe	Po	2	D	AS
2	*S. guianensis*^a^	14-Nov-2017	23°00′47″S; 44°26′32″W (BR)	94	Ca	Ma	Mo	2	D	AS
3	*S. guianensis*^a^	17-Dec-2017	22°56′27″S; 43°59′34″W (BR)	164	Ju	Ma	Mo	3	D	SS
4	*S. guianensis*^a^	17-Dec-2017	23°03′08″S; 44°04′13″W (BR)	93	Ca	Fe	Go	3	D	AS
5	*S. guianensis*^a^	23-Dec-2017	22°58′43″S; 43°57′46″W (BR)	149	Ju	Ma	Mo	2	D	AS
6	*S. guianensis*^a^	25-Dec-2017	23°00′11″S; 43°56′46″W (BR)	125	Ju	Ma	Po	3	D	AS
7	*S. guianensis*^a^	26-Dec-2017	22°59′49″S; 43°55′12″W (BR)	188	Ad	Fe	ND	3	D	AS
8	*S. guianensis*^a^	27-Dec-2017	22°56′47″S; 44°00′35″W (BR)	176	Ad	Ma	Mo	3	D	AS
9	*S. guianensis*^a^	27-Dec-2017	22°56′50″S; 44°02′16″W (BR)	183	Ad	Ma	Mo	3	D	AS
10	*S. guianensis*^a^	27-Dec-2017	23°01′08″S; 43°54′06″W (BR)	186	Ad	Ma	Po	3	D	AS
11	*S. guianensis*^a^	15-Jan-2018	22°56′45″S; 43°54′26″W (BR)	130	Ju	Fe	Po	2	D	SS
12	*S. coeruleoalba*^a^	13-Nov-2002	28°9′2″N; 15°32′8″W (SP)	224	Ad	Ma	Go	2	A	CS
13	*T. truncatus*^a^	18-Jul-2005	29°7′41″N;13°27′58″W (SP)	250	Ju	Fe	Mo	2	A	SS
14	*S. coeruleoalba*^a^	16-Aug-2005	28°0′24″N; 15°22′35″W (SP)	168	Ju	Fe	Go	2	A	AS
15	*S. coeruleoalba*^a^	16-Apr-2007	28°33′28″N; 16°20′1″W (SP)	195	Ju	Ma	Po	2	D	AS
16	*S. coeruleoalba*^a^	02-May-2008	28°29′53″N; 13°50′59″W (SP)	194	Ju	Fe	Po	3	D	BOFDI
17	*S. coeruleoalba*^a^	22-Jan-2009	28°28′2″N; 13°51′37″W (SP)	212	Ad	Fe	Go	2	D	BOFDI
18	*S. coeruleoalba*^a^	10-Feb-2011	28°54′18″N; 13°44′20″W (SP)	215	Ad	Fe	Go	2	D	CS
19	*S. coeruleoalba*^a^	28-Apr-2012	28°55′57″N; 13°49′46″W (SP)	203	Ju	Ma	Mo	2	D	AS
20	*S. coeruleoalba*^a^	04-Jul-2011	40°06′38.5″N 15°13′10.1″E (IT)	205	Ad	Ma	Mo	3	D	BOFDI
21	*S. coeruleoalba*^a^	20-Oct-2013	40°54′15.4″N 14°01′47.3″E (IT)	NR	Ad	Ma	ND	3	D	AS
22	*S. coeruleoalba*^a^	02-Feb-2013	40°38′04.6″N 14°49′47.3″E (IT)	NR	Ad	Fe	ND	3	D	CS
23	*T. truncatus*^a^	20-Mar-2013	43°09′48.9″N 10°32′20.9″E (IT)	203	Ju	Ma	ND	2	D	AS
24	*S. coeruleoalba*^a^	05-Feb-2013	38°12′57.1″N 15°13′50.8″E (IT)	202	Ad	Ma	ND	2	D	CS
25	*T. truncatus*^a^	30-Jun-2011	41°37′32.6″N 12°27′18.2″E (IT)	297	Ad	Ma	Mo	2	A	SS
26	*S. coeruleoalba*^a^	12-Oct-2017	42°28′05″N 14°13′27″E (IT)	200	Ad	Fe	Mo	2	D	AS
27	*S. coeruleoalba*^a^	10-Nov-2017	42°10′37″N 14°41′33″E (IT)	188	Ju	Fe	Mo	3	D	SS
28	*S.coeruleoalba*^b^	29-Apr-09	28.002990, −15.373500 (SP)	105	Ca	Fe	Go	2	D	Not infected
29	*T. Truncatus*^b^	15-Oct-2008	44.006247, 12.662941 (IT)	118	Ca	Ma	Mo	2	D	Not infected
30	*S. guianensis*^b^	26-Nov-17	23°00′57″S; 43°55′23″W (BR)	89	Ca	Ma	Mo	2	D	Not infected

a *CeMV-positive*;

b *CeMV-negative; NR, not recorded; Ca, calf; Ju, juvenile; Ad, adult; Fe, female; Ma, male; NS, nutritional status; Po, poor; Mo, moderate; G, good; DC, decomposition code (2, fresh; 3, moderate autolysis); SC, stranding condition (A: alive; D: dead). AS, acute systemic; SS, subacute systemic, CS, chronic systemic; BOFDI, brain only form of DMV infection*.

Consistent immunolabeling (with variations according to LIR) was detected for CAS3, CD3, CD20, CD57, CD68, FoxP3, MHCII, Iba1, IFNγ, IgG, IL4, IL10, lysozyme, TGFβ, and PAX5 in all organs/tissue sections examined. The following alterations in immunophenotypic profiles of LIRs are referred as to comparisons to “control” animals. “Normal” or “physiologic” antigen cell distribution and intensity for the pAbs aforementioned in control animals (cases 28, 29, and 30) were similar to those previously reported (13) (Supplemental Figure).

### Lymphoid Tissues: Lymph Nodes, Spleen

**Figure 1:**
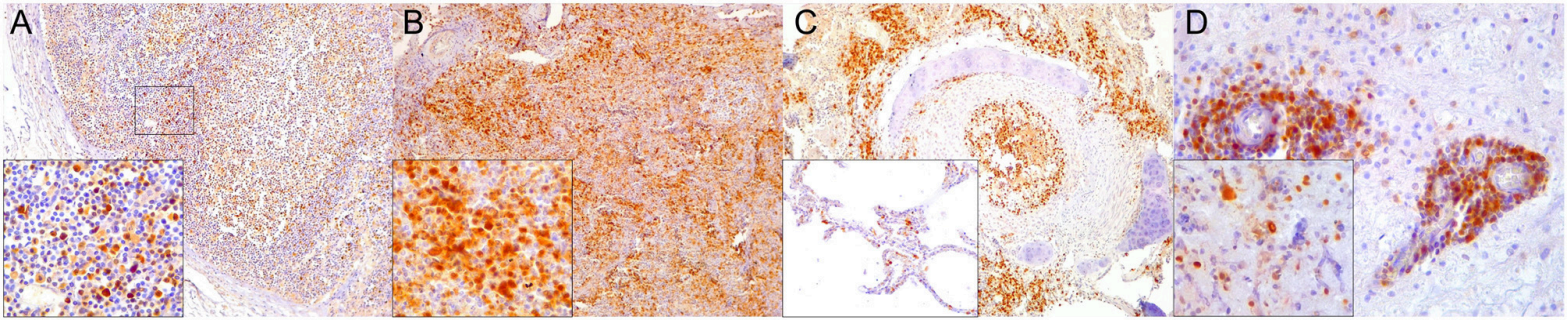
Caspase 3 (CAS3) immunohistochemical findings. **(A)** Lymph node (case 22). Increased expression of CAS3 throughout the cortex. 100×. Inset: lymph node (case 22). Detail of increased CAS3 expression in mononuclear cells (MNCs) of mantle and paracortex. 400×. **(B)** Spleen (case 21). Increased expression of CAS3 throughout the white and red pulp. 100×. Inset: spleen (case 21). Detail of increased expression of CAS3 in white and red pulp. 400×. **(C)** Lung (case 18). Increased expression of CAS3 in proliferative and necrotizing bronchiolitis. 100×. Inset: lung (case 21). Detail of CAS3+ circulating MNCs in alveolar capillaries. 200×. **(D)** Cerebrum (case 24). Increased CAS3 expression in inflammatory cell infiltrates in Virchow-Robins space and adjacent neuroparenchyma. 400×. Inset: cerebrum (case 26). Detail of CAS3^+^ neurons, neuroglia, and infiltrating MNCs. 400×.

**Figure 2:**
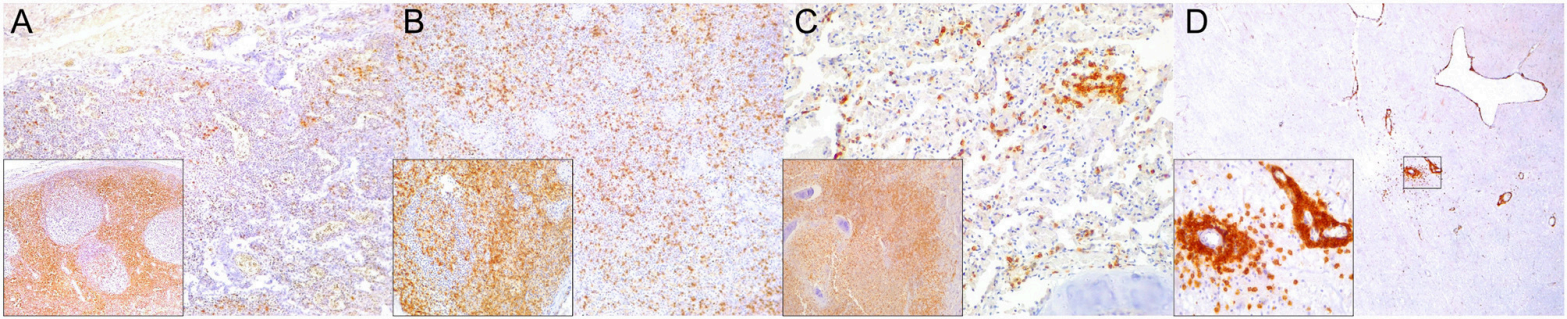
Cluster of differentiation (CD)-3 immunohistochemical findings. **(A)** Lymph node (case 4). Markedly depleted CD3^+^ lymphocytes of cortex and paracortex. 100×. Inset: lymph node (case 22). CD3^+^ lymphocyte hyperplasia in paracortex (interfollicular). 400×. **(B)** Spleen (case 24). Reduced CD3^+^ lymphocytes through white pulp. 100×. Inset: spleen (case 17) CD3^+^ hyperplasia in white pulp and red pulp districts. 200×. **(C)** Lung (case 17). Mild, multifocal circulating and infiltrating CD3^+^ lymphocytes in alveolar walls and interstitium. 200×. Inset: lung (case 25). Marked CD3^+^ lymphocyte bronchiolar hyperplasia. 100×. **(D)** Cerebrum (case 24). Multiple Virchow-Robin spaces are expanded by CD3^+^ lymphocytes, which also infiltrate the adjacent parenchyma. 40×. Inset: cerebrum (case 24). Detail of abundant CD3^+^ lymphocytes perivascular and neuroparenchymal infiltration. 400×.

**Figure 3:**
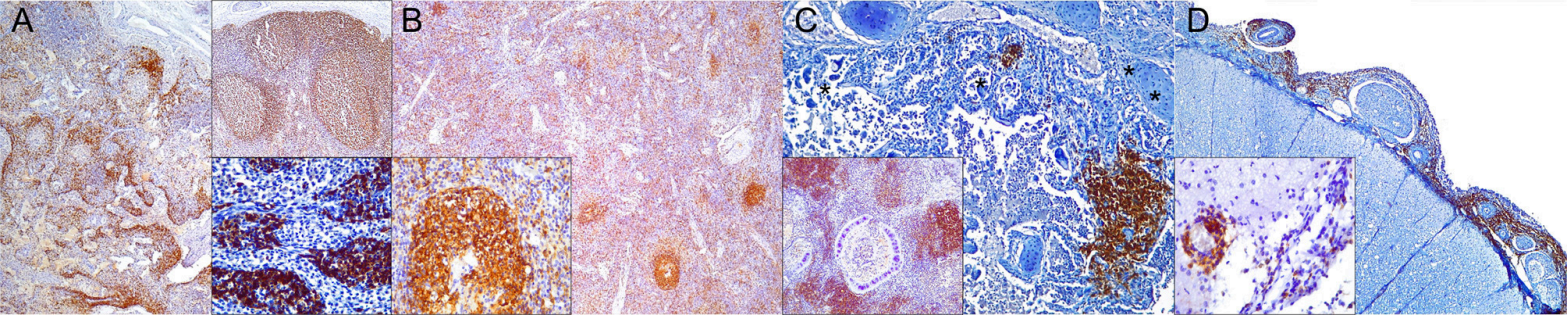
Cluster of differentiation (CD)-20 immunohistochemical findings. **(A)** Lymph node (case 18). Moderate depletion of CD20 lymphocytes in cortical areas. 40×. Upper inset: lymph node (case 22). CD20^+^ lymphocyte hyperplasia. 40×. Lower inset: lymph node (case 18). CD20^+^ lymphocyte hyperplasia in medullary cords. 400×. **(B)** Spleen (case 21). Mild to moderate multifocal CD20-expressing lymphocyte depletion in white pulp. 40×. Inset: spleen (case 21). Detail of relatively normal CD20^+^ lymphocyte number in follicle. **(C)** Lung (case 13). Multifocal parabronchiolar, interstitial/alveolar septa CD20^+^ lymphocytic infiltrates. 100×. Inset: lung (case 25). Marked, multifocal peribronchiolar CD20^+^ lymphocytic infiltrates. 100×. **(D)** Spinal cord (case 13). Marked CD20^+^ leptomeningeal infiltration. 40×. Inset: cerebrum (case 26). Subpial CD20 perivascular and leptomeningeal CD20^+^ infiltrates. 400×.

**Figure 4:**
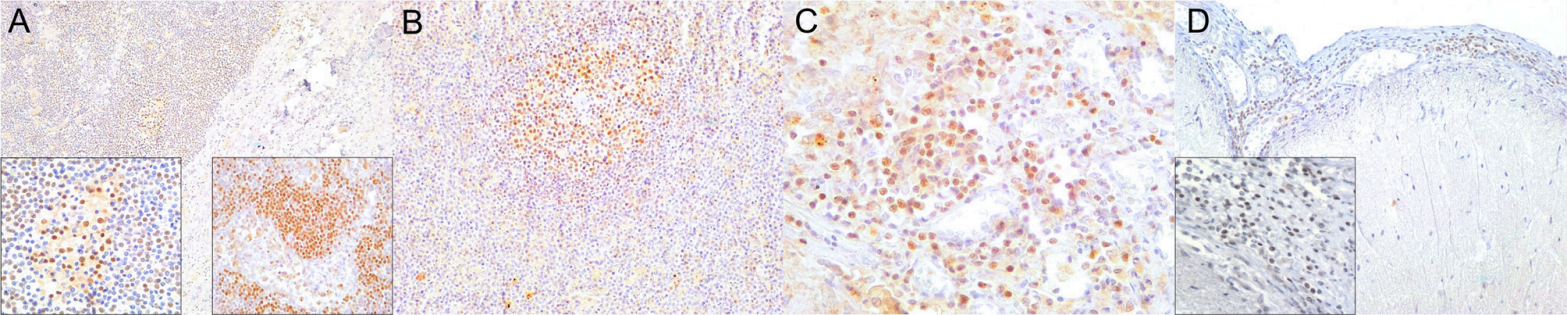
Paired box protein **(**PAX)-5 immunohistochemical findings. **(A)** Lymph node (case 17). Mild to moderate PAX5^+^ lymphocyte depletion in cortical area. 100×. Left inset: lymph node (case 17). Detail of mild PAX5^+^ lymphocyte depletion in follicle. 400×. Right inset: lymph node (case 18). PAX5^+^ lymphocyte hyperplasia in medullary cord. 400×. **(B)** Spleen (case 17). Mild PAX5^+^ lymphocyte follicular depletion. 200×. **(C)** Lung (case 18). Increased numbers of PAX5^+^ lymphocytes in lung interstitium. 400×. **(D)** Cerebrum (case 13). Moderate infiltration of PAX5 lymphocytes in leptomeninges. 200×. Inset: cerebrum (case 13). Detail of leptomeningeal infiltrating PAX5^+^ lymphocytes. 400×.

**Figure 5:**
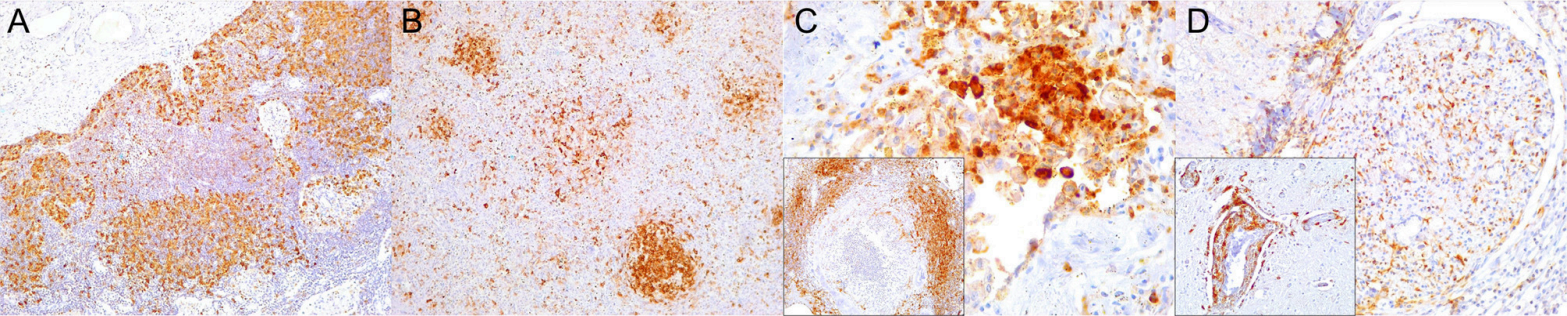
Major histocompatibility (MHC) class II immunohistochemical findings. **(A)** Lymph node (case 12). MHCII expression in cortical cell elements mainly B-cell areas and sinuses. 100×. **(B)** Spleen (case 2). MHCII expression mostly in white pulp cell elements and red pulp. 100×. **(C)** Lung (case 9). Increased expression of MHCII in inflamed alveolus and exudative cells including reactive and binucleated histiocytes and syncytia. 400×. Inset: lung (case 12). Abundant MHCII-expressing inflammatory cells in inflamed distal bronchus. 100×. **(D)** Spinal cord (case 13). Overexpression of MHCII in spinal nerve root cell elements and infiltrating inflammatory cells. 200×. Inset: cerebrum (case 18). Overexpression of MHCII in perivascular inflammatory cells and vascular and neuroparenchymal cell elements. 200×.

**Figure 6:**
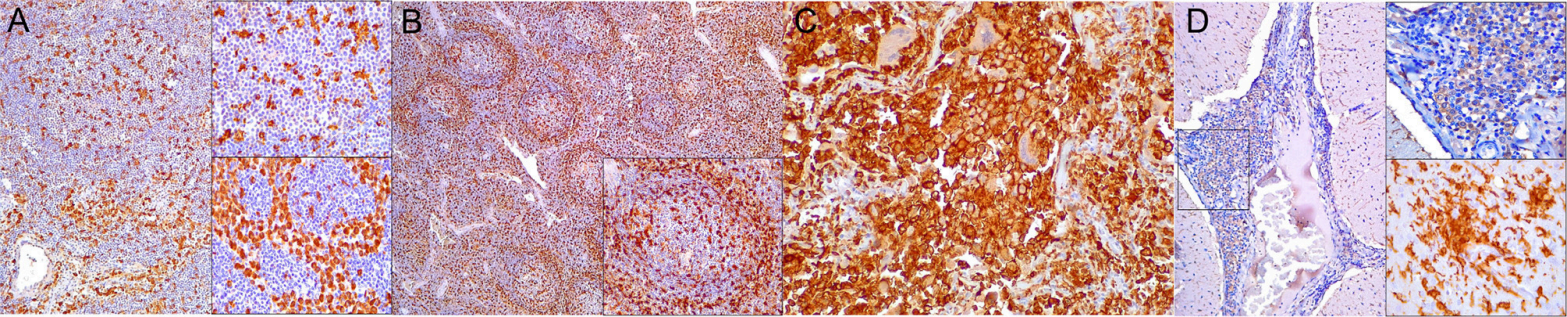
Ionized calcium binding adaptor molecule (Iba)-1 immunohistochemical findings. **(A)** Lymph node (case 17). Small to moderate amounts of Iba1-expressing histiocytes (including intra- and interfollicular dendritic cells, and sinus histiocytes) in cortex/paracortex interface and medullary cords. 100×. Upper inset: lymph node (case 17). Detail of Iba1^+^ follicular dendritic cells. 400×. Lower inset: lymph node (case 18). Detail of Iba1-expressing sinus histiocytes and hyperplastic medullary cords. 400×. **(B)** Spleen (case 15). Abundant expression of Iba1^+^ in histiocytic populations in red and white pulp. 100×. Inset: spleen (case 15). Detail of Iba1-expressing histiocytic cells in follicle and perifollicular zone. 400×. **(C)** Lung (case 19). Striking alveolar Iba1^+^ histiocytic inflammatory response including reactive, binucleated, and multinucleate macrophages/syncytia. 400×. **(D)** Cerebellum (case 25). Marked infiltration of Iba1^+^ histiocytes in interfoliar leptomeninges. 100×. Upper inset: cerebellum (case 25). Detail of Iba^+^ meningeal inflammatory infiltrate. 400×. Lower inset: prominent perivascular histiocytic infiltration including macrophages and microgliosis. 400×.

**Figure 7:**
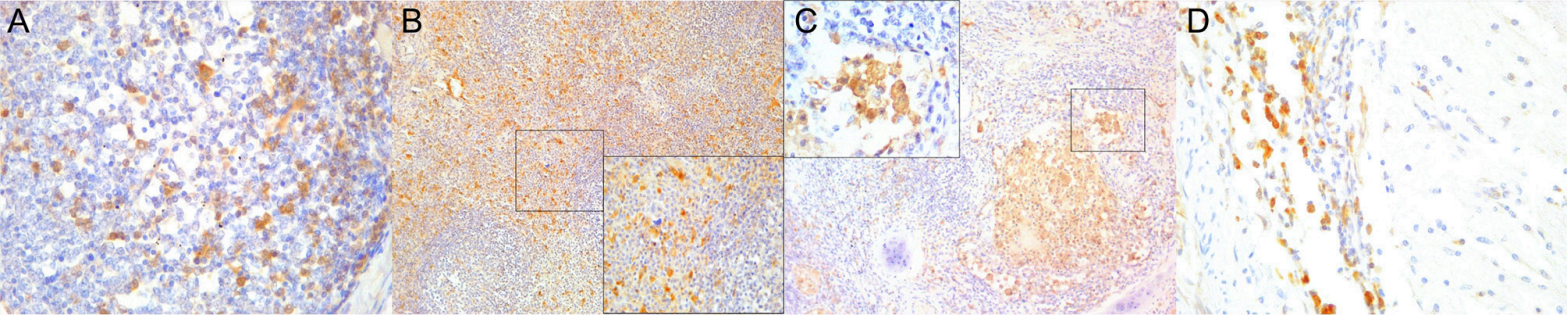
Cluster of differentiation (CD)-68 immunohistochemical findings. **(A)** Lymph node (case 25). Detail of CD68^+^ follicular and interfollicular dendritic (histiocytes) cells. 400×. **(B)** Spleen (case 25). Abundant sinus histiocytic cells expressing CD68. 100×. Inset: spleen (case 25). Detail of perifollicular CD68^+^ histiocytic cells. 400×. **(C)** Lung (case 25). Abundant intrabronchiolar and intraalveolar histiocytes express CD68. Inset: lung (case 25). Detail of intraalveolar histiocytes expressing CD68. 400×. **(D)** Cerebellum (case 25). Detail of CD68^+^ histiocytes infiltrating the leptomeninges. 400×.

**Figure 8:**
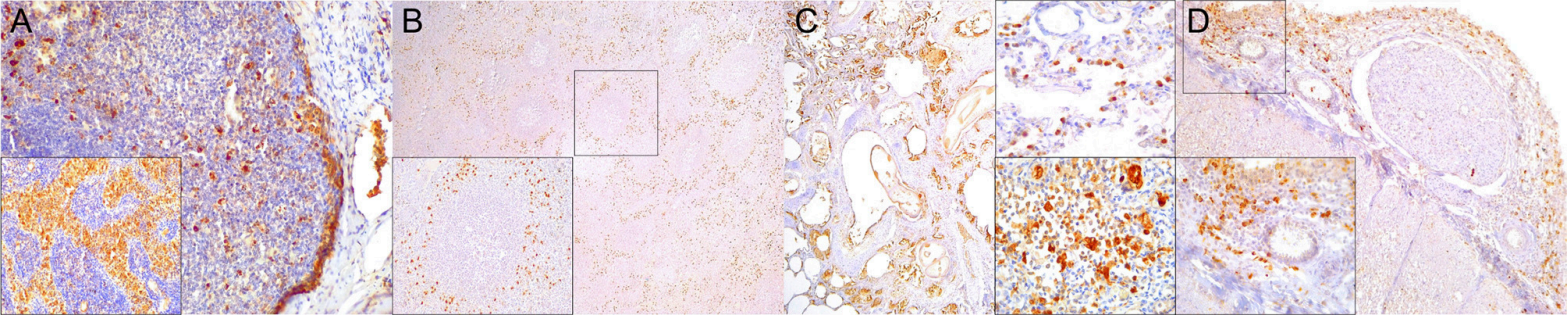
Lysozyme immunohistochemical findings. **(A)** Lymph node (case 25). Lysozyme^+^ histiocytic cells in cortical and paracortical area. 200×. Inset: lymph node (case 25). Abundant intrasinus lysozyme-expressing histiocytes. 200×. **(B)** Spleen (case 14). Lysozyme^+^ histiocytes through the red pulp. 40×. Inset: spleen (case 14). Detail of lysozyme histiocytes in red pulp. 400×. **(C)** Lung (case 11). Overexpression of lysozyme by inflammatory cells and exudate in inflamed bronchioles and alveoli associated with CeMV and concomitant verminous pneumonia. 40×. Upper inset: lung (case 13). Circulating MNCs (including presumed pulmonary intravascular macrophages) are lysozyme^+^. 400×. Lower inset: lung (case 21). Increased lysozyme+ (mainly histiocytes) cells within inflammatory focus. 400×. **(D)** Spinal cord (case 13). Moderate number of lysozyme+ histiocytes in spinal cord leptomeninges. 100×. Inset: spinal cord (case 13). Detail of lysozyme+ histiocytic meningeal infiltrate. 400×.

**Figure 9:**
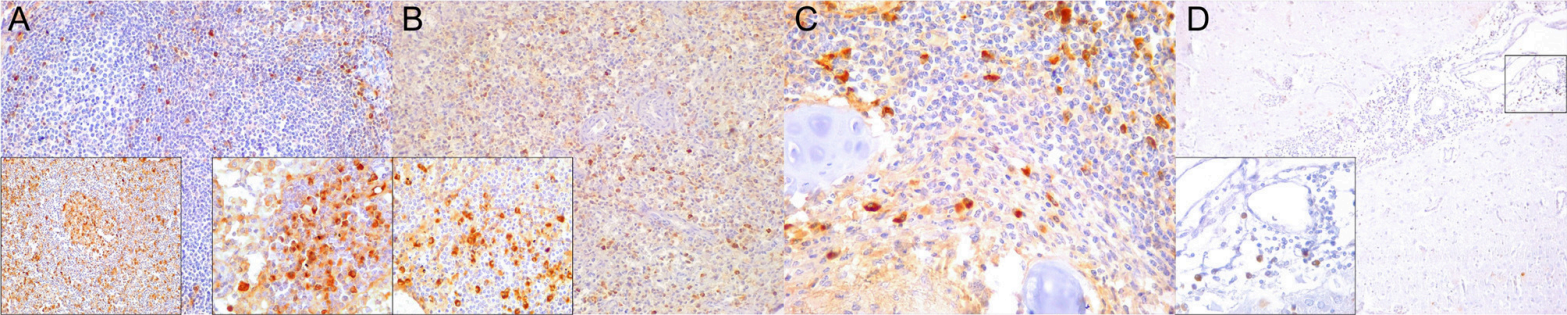
IgG immunohistochemical findings. **(A)** Lymph node (case 18). Scattered IgG^+^ cells in follicular and parafollicular areas. 200×. Left inset: lymph node (case 15). Increased IgG-expressing cells in follicular and parafollicular areas. 200×. Right inset: lymph node (case 12). IgG^+^ cell hyperplasia in medullary cord. 400×. **(B)** Spleen (case 13). Scattered IgG+ cells at white/red pulp interface. 200×. Inset: spleen (case 17). Increased IgG^+^ cells in white/red pulp interface. 400×. **(C)** Lung (case 12). IgG^+^ cells in hyperplastic BALT of inflamed bronchiole. 400×. **(D)** Cerebrum (case 14). Scattered IgG^+^ cells in leptomeninges. 400×. Inset: cerebrum (case 14). Detail of IgG^+^ cells in meningeal infiltrates. 400×.

CeMV-LIR often overlapped focally with verminous lymphadenitis-associated LIR, especially in Guiana dolphins. The following changes had somewhat similar distribution patterns in all lymph nodes (mediastinal/tracheobronchial, pulmonary, mesenteric, prescapular) and spleens evaluated (Table 3). There was an overall increased CAS3 expression in mononuclear cells (MNCs), including lymphocytes and histiocytes (often with engulfed apoptotic debris) in the cortex and paracortex, and histiocytes of subcortical sinuses and medullary cords (Figure 1A) in lymph nodes of CeMV-infected dolphins. While there was a consistently decreased number of CD3+ (Figure 2A), CD20+ (Figure 3A), and PAX5+ (Figure 4A) lymphocytes (hereafter, T cells, and B cells, respectively) in lymph nodes of most Guiana dolphins, striped dolphins, and bottlenose dolphins often had cortical, paracortical, and medullary cord expansion of T cells and B cells in addition to milder multifocal depletion phenomena. MHCII expression varied between CeMV-infected and uninfected animals. Approximately half of the dolphins, more predominantly in Guiana dolphins, infected by CeMV had reduced MHCII expression associated with diminished B-cells; however, there were increased MHCII+ histiocytes (see below) influx (Figure 5A). Fewer MHCII+ MNCs were detected in the paracortex and subcortical medullary cords. Iba1+ histiocytes (macrophages, monocytes, dendritic cells) (Figure 6A) correlated with CD68+ (Figure 7A) and lysozyme+ (Figure 8A) histiocytes; however, Iba1+ tended to label a greater number of histiocytic cells. Increased histiocytes (dendritic cells and circulating monocytes/macrophages) were common in depleted follicles, paracortex, and sinuses of infected dolphins. Occasional syncytia were Iba1+ and CD68+. CD57+ MNCs in lymph nodes varied slightly among CeMV-infected dolphins but overall did not differ considerably from “control” dolphins, with only few scattered immunoreactive cells in cortex and paracortex. Furthermore, the number of IgG+ lymphocytes varied among CeMV-infected dolphins; however, they tended to be slightly more numerous than in “control” animals, being mostly detected in germinal centers (Figure 9A) and paracortex and to a lesser extent in medullary cords and occasionally within sinuses. Scattered FoxP3+ lymphocytes were seen in the cortex, paracortex, and medullary cords of CeMV-infected and control dolphins with no apparent differences from “control” dolphins.

**Table 3:** Summary of the results for selected immunomarkers in lymph nodes and spleen of striped dolphins (*Stenella coeruleoalba*), bottlenose dolphins (*Tursiops truncatus*), and Guiana dolphins (*Sotalia guianensis*) included in this study.

**No**	**Organ**	**CAS3**	**CD3**	**CD20**	**CD57**	**CD68**	**FoxP3**	**HLA-DRα**	**Iba1**	**IFNγ**	**IgG**	**IL4**	**IL10**	**Lysozyme**	**TGFβ**	**PAX-5**
1	LNs	++/3	+/3	+/3	–/0	++/2	+/2	++/3	+/3	+/3	+/3	+/3	+/2	++/3	+/2	+/2
	Spleen	+++/3	+/3	+/3	+/3	++/3	–/0	+,++/3	++/3	+/3	++/3	+/2	++/2	++/3	+/2	+/2
2	LNS	++/2	+/3	+,++/3	–/0	+,++/2	+/2	++,+++/3	+/3	+/3	+/3	+/2	+/2	++/3	+/3	+,++/2
	Spleen	+/2	+/3	+,++/3	+/2	+,++/2	–/0	++/3	++/3	+/2	+/3	+/3	+/2	++/3	+/3	+,++/3
3^a^	LNs	+/2	+/3	+/3	+/3	++/3	+/2	+,++/3	+/2	++/2	+/3	+/2	+,++/2	+,+++/3	+/2	+/2
	Spleen	+/2	+/2	+/3	–/0	+/2	–/0	+/3	+,++/3	+/2	+/2	+/3	+/2	++/3	+/3	+/2
4	LNs	+++/2	+/3	+/3	+/2	+,++/2	+/3	++,+++/3	+,++/3	+/2	+/2	+/2	+/2	++,+++/3	+/2	+/2
	Spleen	+/2	+/3	+/3	–/0	+,++/2	+/2	++/3	++/3	NE	NE	NE	NE	+,++/3	NE	+/2
5	LNs	+++/3	++/3	+,++/3	–/0	+/2	+/3	++/3	+,++/3	+/2	+,++/3	+,++/3	+,++/2	++/3	+/3	+/3
	Spleen	+/2	+/3	+,++/3	–/0	++/3	+/2	+,++/3	+++/2	+/3	+,++/2	+/2	+,++/2	++,+++/3	+/2	+,++/2
6	LNs	+/2	+/2	+/3	+/3	+/3	+/2	++/3	++/2	+/2	+/2	+/2	+/2	+/3	+/2	+/2
	Spleen	+/2	+/2	+/3	+/2	+/3	+/2	+,++/3	+,++/3	–/0	+/2	+/2	+/2	++/3	+/2	+/3
7	LNs	+/3	+/3	+/3	NE	NE	+/2	NE	+,++/3	NE	NE	+/2	+/2	NE	NE	+/3
	Spleen	+/3	+/2	+/3	NE	NE	+/2	NE	+,++/3	NE	NE	NE	NE	NE	NE	+/3
8	LNs	+/3	+/3	+/3	+/2	++/3	+/2	+,++/3	+/3	+/3	+/3	+/3	+/3	+,++/3	+/2	+/3
	Spleen	++/3	+/3	+/3	+/2	++/3	–/0	++/3	+/3	+/2	+/2	+/3	+/2	++,+++/3	+,++/2	+/3
9	LNs	++/3	++/3	+,++/3	+/3	++/3	+/3	++/3	+/3	NE	+/3	+/3	NE	++,+++/3	NE	+/2
	Spleen	+/2	++/2	+,++/3	+/3	++/2	+/3	+/2	+/3	+/3	+/3	+/2	+/2	++/3	+/2	+,++/2
10	LNs	+++/3	++/3	+,++/3	+/2	++/2	+/3	+,++/3	+/3	+/2	+/3	+/2	+/3	++/3	+/2	+,++/2
	Spleen	+/2	++/3	+/3	+/2	++/3	+/2	+/3	++/2	+/2	+/3	+,++/2	+,++/2	++,+++/3	+/2	+/2
11^b^	LNs	+/2	+/3	+/3	+/2	+/3	+/2	+/3	+/3	+3	+,++/3	+/3	+/2	++/3	+/2	+/2
	Spleen	+/2	+/3	+/3	–/0	++/3	–/0	+/3	+,++/3	+/3	+,++/3	+/2	+,++/3	+/3	+,++/2	+/2
12	LNs	+/3	++,+++/3	+,++/3	+/3	+,++/2	+/3	++/3	++,+++/3	+/3	++,+++/3	+/3	+/2	+,++/3	+/3	+,++/3
	Spleen	++/2	++/2	++/3	+/3	+,++/3	+/3	++,+++/2	++,+++/3	+,++/3	+,++/3	+/2	+/3	++/3	+/2	+/3
13^c^	LNs	++/3	+/3	+,++/3	+/3	++/2	+/3	++,+++/3	++,+++/3	+/3	++/3	+/2	+/3	+,++/3	+/2	+/2
	Spleen	++/2	+/3	+/3	+/2	+,++/3	+/3	++/3	++,+++/3	+/3	++/3	+/3	+/3	++/3	+/3	+/2
14	LNs	+++/2	+++/2	++/3	+/2	+,++/2	+/3	++,+++/3	++,+++/3	+/3	+/2	+/2	+/2	+,++/3	+/2	++/2
	Spleen	++/3	++,+++/3	++/3	+/2	+,++/2	+/3	++,+++/3	++,+++/3	+/3	+/3	+/2	+/2	++/3	+/2	++/2
15	LNs	+++/3	+,++/3	+,++/3	+/3	++/2	+/3	++/3	++/3	+,++/3	+,++/3	+/3	+/3	++/2	+,++/2	+,++/2
	Spleen	+++/3	++/2	++/3	+/3	+/2	+/3	++/3	++,+++/3	+,++/3	++/3	+/2	+/3	++/2	+/2	++/2
16	LNs	++/3	+/2	+,++/3	+,++/3	+/3	+/2	+,++/3	++/3	+,++/2	++/2	+/2	+/3	+,++/2	+/3	+,++/2
	Spleen	++/3	++/3	+,++/3	+/3	+,++/2	+/2	+,++/3	++/3	+/3	+/3	+/3	+/2	+/2	+/2	+,++/2
17	LNs	+++/3	++/3	++,+++/3	+/3	+,++/3	+/3	+,++/3	++/3	+/3	+,++/3	+/3	+/3	+,++/3	+/3	+,+++/3
	Spleen	++/3	++/3	+,++/3	–/0	+,++/2	+/3	++/3	++/3	+/3	++/3	+/3	+/3	+,++/3	+/2	+,++/3
18	LNs	++/3	++/3	+,++/3	+/3	+,++/2	+/3	++/3	++/3	+,++/3	+/3	+/3	+/3	++/3	+/2	+,+++/3
	Spleen	++/3	++,+++/3	+,++/2	+/2	+/2	+/3	++/3	++/3	+/3	+,++/3	+/2	+/2	++/2	+/2	+,+++/3
19	LNs	+++/2	+++/2	+,++/3	++/3	++3	+/3	++,+++/3	++,+++/3	+,++/3	+/3	+/3	+,++/3	++,+++/3	+/2	++/2
	Spleen	++/2	++/3	+/2	+,++/3	+,++/3	+/3	++,+++/3	++,+++/3	+/3	+,++/3	+/2	++/2	++/3	+/2	+/2
20	LNs	+/3	+/3	+,++/3	+/2	+,++/2	–/0	+,++/3	++/3	NE	NE	NE	NE	NE	NE	+/2
	Spleen	+/3	++/2	++/3	–/0	+/2	+/3	+,++/3	++/2	+/2	+/2	+/2	+/2	++/3	+/2	+/2
21	LNs	++/3	++/2	++/3	+/2	NE	+/3	+,++/3	++/3	NE	NE	NE	NE	NE	NE	++/2
	Spleen	+++/3	++,+++/2	++,+++/3	+/3	++/3	+/2	+,++/3	+,++/3	+/3	+/3	+,++/2	+/3	++/3	+,++/2	++/2
22	LNs	++/3	+++/3	++,+++/3	+/2	++/2	+/3	++/3	+,++/2	+/2	++,+++/3	+/3	+/2	+ to ++/3	+/2	++,+++/3
	Spleen	++/3	++/3	++,+++/3	+/2	+,++/2	+/3	++/3	+,++/2	++/3	++/3	+/3	+/2	++/3	+/2	++/2
23	LNs	++/2	+++/2	++,+++/3	+/3	+/3	+/2	++/2	++/3		+/2	+/2	+/2	+/3	+/2	++/2
	Spleen	++/2	++/2	++/3	+/2	NE	+/2	+,++/2	++,+++/2	+,++/2	+/2	NE	NE	++/3	NE	++/2
24	LNs	++/2	++/3	++/3	+/3	++,+++/2	+/2	+,++/3	++/3		NE	+,++/2	NE	NE	NE	++/3
	Spleen	++/2	++/3	++/3	+/3	+,++/3	+/3	+,++/3	+++/2	+/3	+,++/3	+/3	+/2	++/3	+/3	++/3
25^d^	LNs	++/3	++,+++/3	+,++/3	+,++/3	++,+++/2	+/3	++/3	++/2	+,++/2	++/3	++/2	+/3	++/+++/3	+/3	+,++/2
	Spleen	+++/3	++/2	++/3	+/3	++,+++/3	+/3	++/3	++/3	+/2	+/3	+/2	+/3	++/3	+/2	++/2
26	LNs	+/3	+/3	+/3	NE	NE	+/2	++/3	++/3	NE	NE	NE	NE	NE	NE	+/3
	Spleen	+/3	+/3	++/3	NE	NE	+/2	++/3	++/3	NE	NE	NE	NE	NE	NE	+/3
27^e^	LNs	NE	NE	NE	NE	NE	NE	NE	NE	NE	NE	NE	NE	NE	NE	NE
	Spleen	+/3	+/3	++/3	NE	NE	+/2	++/3	++,+++/3	NE	NE	NE	NE	NE	NE	NE
28	LNs	+/3	++,+++/3	++,+++/3	+/3	–/0	+/3	++,+++/3	++,+++/3	–/0	+/2	+/2	+/2	+/3	–/0	++,+++/2
	Spleen	+/3	++/3	++,+++/3	+/2	+/2	–/0	+,++/3	++/2	–/0	+/2	+/2	+/2	+/2	+/2	++,+++/2
29	LNs	+/3	++,+++/3	++,+++/3	++/2	–/0	+/2	++,+++/3	++/3	+/3	+/2	–/0	+,++/2	+/3	+/2	++,+++/2
	Spleen	+/3	++/3	++,+++/3	+/2	–/0	–/0	+,++/3	++/3	+/3	+/2	+/2	+/2	+/2	+/2	++,+++/2
30	LNs	+/3	++,+++/3	++,+++/3	+/2	+/2	+/3	++/3	++/3	+/3	+/2	+/2	+,++/2	+/3	+/3	++,+++/2
	Spleen	+/3	++/3	++,+++/3	+/2	+/2	+/2	++,+++/3	++/3	+/2	+/2	+/2	+/2	+/2	+/2	++,+++/2

*CD, cluster of differentiation; FoxP3, Forkhead Box P3; HLA, human leukocyte antigen (synonym major histocompatibility complex); IFN, interferon; Ig, immunoglobulin; IL, interleukin; TGF, transforming growth factor; Pax-5, paired box 5; NE, not evaluated. Semiquantitative analysis of immunopositive cells: –, no; +, <10%; ++, 10–50%; +++, 50–90%, and ++++, >90% immunopositive cells. Subjective labeling intensity score of immunopositive cells: 0, 1, 2, and 3*.

a *Multisystemic hyphate mycosis (lung, kidney)*.

b *Hyphate mycosis (lung)*.

c *Suspect Brucella coinfection (primarily CNS lesions)*.

d *Sepsis by Staphylococcus aureus (particularly severe CNS and lung lesions)*.

e *Multisystemic hyphate mycosis (lung, cerebrum)*.

In the spleen, there was increased CAS3 expression in MNCs of white pulp and to a lesser extent in red pulp (Figure 1B). There was a consistently reduced number of T cells (Figure 2B) and B cells (Figures 3B, 4B), especially prominent in Guiana dolphins; however, striped dolphins and bottlenose dolphins occasionally presented “reactive (regenerative) hyperplasia” in addition to multifocal lymphoid depletion. As observed in lymph nodes, infected dolphins tended to have reduced expression of MHCII in B cell areas (Figure 5B); however, increased MHCII+ histiocytes contributed to overall similar semiquantitative results for MHCII expression in spleen of “control” animals. Iba1+ (Figure 6B) histiocytes correlated to CD68+ (Figure 7B) and lysozyme+ (Figure 8B) histiocytes, and were more numerous in the red pulp. Rare CD57+ MNCs were detected in the white pulp with no evident difference regarding “control” dolphins. IgG+ lymphocytes were more common in the white pulp and to a lesser extent in the red pulp (Figure 9B) of infected dolphins, particularly in striped dolphins from the Canary Islands and Italy, in contrast to Guiana dolphins. No differences were detected regarding FoxP3+ lymphocytes in CeMV-infected and uninfected “control” dolphins.

**Figure 10:**
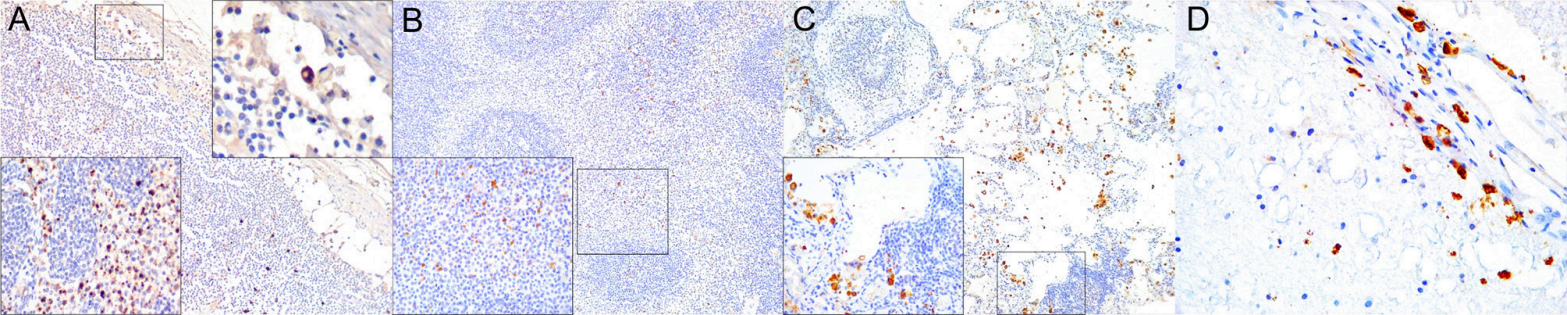
Interferon (IFN)-γ immunohistochemical findings. **(A)** Lymph node (case 17). Scattered MNCs including lymphocytes and histiocytes express IFNγ in cortex, paracortex and subcapsular/marginal sinus (square). Right inset: lymph node (case 17). IFNγ-expressing histiocyte within subcapsular/marginal sinus. 400×. Left inset: lymph node (case 17). Moderate number of IFNγ-expressing histiocytes and lymphocytes within paracortical/medullary sinuses. **(B)** Spleen (case 22). Scattered IFNγ-expressing cells in white and red pulp. Detail of IFNγ-expressing cells. 100×. Inset: 22. 400×. **(C)** Lung (case 23). IFNγ-expressing cells mainly include alveolar macrophages and scattered interstitial mononuclear cells. 100×. Inset: lung (case 23). Detail of IFNγ-expressing cells within perialveolar lymphocytic aggregate and rare alveolar macrophage. 400×. (D) Cerebrum (case 9). Meningeal and neuroparenchymal IFNγ-expressing cells. 400×.

Approximately a third of the infected dolphins had increased IFNγ immunoreactivity in lymph nodes and/or spleen tissue compared to uninfected dolphins. IFNγ was variably expressed in MNCs (lymphocytes, macrophages), rare syncytia and extracellularly in lymph nodes, and spleen (Figures 10A,B). Except for few dolphins, most infected dolphins showed no immunohistochemically evident difference regarding TGFβ, IL4, and IL10 immunoreactivity in lymph nodes and spleen. Occasional MNCs expressed TGFβ, rarely expressed also by syncytia (case 25). Scattered IL4+ and IL10+ MNCs along with occasional extracellular labeling were noted in cortex and paracortex (lymph nodes) and white pulp (spleen), and to a lesser extent in lymph node and spleen sinuses.

### Lung

CeMV-LIR often overlapped focally with verminous pneumonia-associated LIR, especially in Guiana dolphins. IHC results in lung tissue are recorded in Table 4. There was increased CAS3 expression in circulating, infiltrating, and exocytosing/exudating MNCs as well as luminal cell debris (Figure 1C). There was a mildly to moderately increased number of T cells (Figure 2C) and B cells (Figures 3C, 4C) in alveolar septa, interstitium, and bronchial/bronchiolar mucosa/submucosa including BALT. Lung from “control” dolphins had scattered detectable T cells and B cells. Case 25 (with concomitant septicemia by *Staphylococcus aureus*) had abundant T cell and B cell infiltrates around bronchi/bronchioles, and alveoli (Figure 2C). Overall, the number and intensity of CAS3+ and T cells and B cells was greater in parasitic inflammatory foci. MHCII immunoexpression was consistently increased and involved presumed resident and circulating MNCs, including putative pulmonary intravascular macrophages (PIVMs), and infiltrating inflammatory MNCs (Figure 5C), being more abundant in foci of parasitic injury. Histiocytes were increased in CeMV-infected dolphins. Iba1+ histiocytes were seen circulating as well as infiltrating the bronchial/bronchiolar mucosa/submucosa, BALT, interstitium, and exocytosing and filling the alveolar lumina (Figure 6C). CD68+ (Figure 7C) and lysozyme+ (Figure 8C) histiocytes paralleled Iba-expressing cells yet they were often less numerous. Histiocytes and lysozyme+ neutrophils were overall more abundant in parasitic inflammatory foci. Hyperplastic (type II) pneumocytes and alveolar exudates expressed lysozyme in many animals, including parasitic and fungal pneumonia cases. CD57+ MNCs were rare in the lung; no differences were noted compared to control animals. IgG+ lymphocytes were increased in CeMV-infected dolphins and were detected mainly in BALT (Figure 9C) and to a lesser extent in alveolar septa and interstitium; they were especially prominent in parasitic LIR. Intra-alveolar syncytia were generally Iba1+. Rare FoxP3+ lymphocytes were seen in lung of some CeMV-infected dolphins; by contrast, no FoxP3-expressing lymphocytes were seen in control dolphins.

**Table 4:** Summary of results for selected immunomarkers in lung of Guiana dolphins (*Sotalia guianensis*), striped dolphins (*Stenella coeruleoalba*), and bottlenose dolphins (*Tursiops truncatus*) included in this study.

**No**	**CAS3**	**CD3**	**CD20**	**CD57**	**CD68**	**FoxP3**	**HLA-DRα**	**Iba1**	**IFNγ**	**IgG**	**IL4**	**IL10**	**Lysozyme**	**TGFβ**	**PAX-5**
1	+/2	+/2	+/3	–/0	+/2	–/0	+/3	+/3	+/2	+,++/3	+/3	+/2	+/3	+/2	+/2
2	+/2	+/3	+/3	+/3	+/3	–/0	+/3	+/3	+/3	+,++/3	+/3	+/2	+/3	+/2	+/2
3^a^	++/2	+/3	+/3	–/0	++/2	+/2	+,++/3	+/3	+,++/2	+,++/3	+/3	+,++/2	++,+++/3	+/2	+/2
4	+/2	+/3	+/3	–/0	++/2	–/0	+/3	+/3	++/3	+/3	+/3	+/2	+/3	+/2	+/2
5	+/3	+/3	+/3	–/0	+/2	+/2	+/3	++/3	+/3	+/2	+/2	+,++/2	++/3	+/2	+/3
6	+/2	+/2	+/3	+/3	+/3	–/0	+/3	+/2	+/2	+/3	+/2	+/2	+/3	+/2	+/2
7	+/2	+/3	+/3	+/2	+/3	+/2	+/3	+/2	+/3	+/2	+/2	+/2	+/3	+/2	+/2
8	+/2	+/3	+/3	–/0	+/3	+/3	+/2	+/3	+/3	+,++/3	+/2	+/2	+,++/3	+/2	+/2
9	++/3	++,+++/3	+/3	–/0	+/3	+/3	++/3	++/3	+/2	+,++/3	+,++/2	+,++/2	+/3	+/2	+/2
10	++/2	+/3	+/3	–/0	+/3	+/3	+/3	+/3	+/2	+/3	+/2	+/2	++/3	+/2	+/2
11^b^	++/2	+/2	+/3	–/0	+/2	+/2	+/3	++/3	+/3	+/3	+,++/2	++,+++/3	++,+++/3	+/2	+/2
12	+/3	+/3	+,++/3	+/3	++/3	+/3	+,+++/3	++/3	+/3	++/3	+/3	+,++/3	+/3	+/3	+/3
13^c^	++/3	+/2	+,++/3	–/0	++/3	+/2	+,++/3	++/3	+/3	+/3	+/3	++,+++/3	++,+++/3		++/2
14	++/3	+/2	+/2	–/0	+/3	+/2	+/3	+,++/3	+/3	+/2	+/2	+/3	+/3	+/2	+/2
15	+/3	+/3	+,++/3	+/3	+/3	+/3	++/3	+,++/3	+,++/3	+,++/3	+/3	++,+++/3	++/2	+/2	+/3
16	++/3	++/3	+/3	–/0	+/3	+/2	+/3	+,++/3	+/3	+/2	+/2	+/2	+/3	+/2	+/3
17	++/3	++/3	++/3	+/2	+/3	+/3	+,++/3	+,++/3	+,++/3	+/3	+/3	+,++/2	+,++/2	+/3	+/2
18	++/3	++/3	++/3	+/3	++/3	+/3	++,+++/3	++/3	+/3	++/3	+/2	+,++/3	++/3	+/2	+,++/3
19	+++/3	++/3	+,++/2	+/2	+/2	+/3	+,++/3	++++/3	+++/3	++/3	+,++/3	++,+++/2	+,++/3	+/3	+/2
20	+/3	+/2	+/3	–/0	+/2	+/3	+/3	+,++/3	+/3	+/3	+/3	+/3	+,++/3	–/0	+/2
21	+/3	+/3	+/3	+/2	+/2	+/2	+/3	+/3	+/3	+/3	+/2	+/3	+,++/3	+/2	+/2
22	++/3	+,++/3	+/3	+/2	+/2	+/2	+,+++/3	+/3	+/2	+/2	+,++/2	+/2	+/3	+/2	+/3
23	+/2	+/3	+/3	+/2	+/3	+/2	+,++/3	+/3	+,++/3	+/3	+/2	+/3	+/3	+/2	+/2
24	+/3	+/3	+/3	–/0	+/3	+/2	+/3	+/3	+/2	+/3	+/3	+/3	+/3	+/2	+/2
25^d^	++/3	++++/3	++,+++/3	+/3	++,+++/3	+/3	+,++/3	+/3	+/3	+,++/3	++,+++/2	+/3	++,+++/3	+/2	+,++/3
26	+/3	+/3	+/3	NE	NE	NE	NE	+/3	+/3	+/3	NE	NE	NE	NE	NE
27^e^	+/3	+/2	+/3	–/0	+,++/3	+/2	+/2	+/2	+/2	+/3	+/2	+/2	+,++/3	+/2	+/2
28	+/3	+/3	+/3	–/0	–/0	–/0	+/3	+/3	–/0	–/0	+/2	+/2	–/0	–/0	+/3
29	+/3	+/3	+/3	–/0	–/0	–/0	+/3	+/3	+/3	+/2	+/2	+,++/2	–/0	+/3	+/3
30	+/3	+/3	+/3	–/0	–/0	–/0	+/3	+/3	–/0	+/2	+/2	+/2	+/2	+/2	+/3

*CD, cluster of differentiation; FoxP3, Forkhead Box P3; HLA, human leukocyte antigen (synonym major histocompatibility complex); IFN, interferon; Ig, immunoglobulin; IL, interleukin; TGF, transforming growth factor; Pax-5, paired box 5; NE, not evaluated. Semiquantitative analysis of immunopositive cells: –, no; +, <10%; ++, 10–50%; +++, 50–90%, and ++++, >90% immunopositive cells. Subjective labeling intensity score of immunopositive cells: 0, 1, 2, and 3*.

a *Multisystemic hyphate mycosis (lung, kidney)*.

b *Hyphate mycosis (lung)*.

c *Suspect Brucella coinfection (primarily CNS lesions)*.

d *Sepsis by Staphylococcus aureus (particularly severe CNS and lung lesions)*.

e *Multisystemic hyphate mycosis (lung, cerebrum)*.

All “control” dolphins had scattered MNCs immunoreactive and multifocal extracellular signal for IFNγ, IL4, IL10, and TGFβ in lung tissue. However, even though these cytokines appeared expressed by a slightly greater number of MNCs in lung of infected dolphins they represented <10% of the inflammatory cells (including BALT and interstitium); thus, no IHC evident differences were noted based on the proposed semiquantitative approach. IFNγ was mainly expressed by exudating MNCs including alveolar macrophages and syncytia (Figure 10C), and fewer infiltrating macrophages and lymphocytes, and occasional epithelial cells with an extracellular signal in inflammatory foci. Both, IL4 and IL10 were expressed by infiltrating and circulating MNCs compatible with histiocytes and lymphocytes, and extracellularly. TGFβ+ cells were less numerous and typically involved alveolar macrophages, bronchial/bronchiolar epithelia, exocytosing MNCs, and occasional mesenchymal cells.

### Central Nervous System

IHC results are recorded in Table 5. CAS3+ MNCs were seen in meningeal, parenchymal, Virchow-Robin spaces (VRSs), and neuroparenchymal inflammatory infiltrates as well as in circulating MNCs (Figure 1D). In areas of marked neurodegeneration, neurons and neuroglia occasionally expressed CAS3. In decreasing order, T cells (Figure 2D) and B cells (Figures 3D, 4D) predominated in meningeal, VRSs, and neuroparenchymal inflammatory infiltrates in striped dolphins and bottlenose dolphins with varying degrees of CNS inflammation. B-cells were comparatively more numerous in case 13, a case with a suspect *Brucella* co-infection based on cellular inflammatory components and neuroanatomical distribution of lesions. Rare T cells were seen circulating and/or in meningeal perivascular spaces in three Guiana dolphins, likely representing early CNS inflammation. CAS3+ cells appeared to involve mostly T and B cells. MHCII was widely overexpressed, involving MNCs either circulating or expanding the VRS and/or infiltrating the neuroparenchyma, along with vascular endothelial cells (Figure 5D). Occasionally, neuroglia (microglia and astrocytes) expressed MHCII. Few animals additionally exhibited MHCII labeling in roots of spinal nerves and meningeal mesenchyme. Inflamed CNS tissues often harbored Iba1+ histiocytes, either circulating or located within the vessel walls, expanding the VRSs and infiltrating the neuroparenchyma (Figure 6D). Microglia was consistently labeled by Iba1. In few cases, degenerating neurons expressed Iba1+. Lysozyme+ MNCs were rare in the CNS, except for cases 13 and 25 (Figure 7D). CD68+ MNCs were rarely seen in CNS inflammatory foci (Figure 8D). CD57+ cells were not detected in CNS tissues examined. Small numbers of IgG+ lymphocytes were common in meningeal inflammatory infiltrates and VRS (Figure 9D) of striped dolphins. Case 13, a bottlenose dolphin with a suspect coinfection by *Brucella* sp. had greater numbers of IgG-expressing cells and histiocytes. Only cases 13 and 14 showed rare FoxP3+ cells intermingled with perivascular inflammatory infiltrates in CNS tissue sections examined.

**Table 5:** Summary of results for selected immunomarkers in cerebrum, cerebellum, brain stem, and spinal cord of Guiana dolphins (*Sotalia guianensis*), striped dolphins (*Stenella coeruleoalba*), and bottlenose dolphins (*Tursiops truncatus*) included in this study.

**No**	**CAS3**	**CD3**	**CD20**	**CD57**	**CD68**	**FoxP3**	**HLA-DRα**	**Iba1**	**IFNγ**	**IgG**	**IL4**	**IL10**	**Lysozyme**	**TGFβ**	**PAX-5**
1	+/2	–/0	–/0	–/0	–/0	–/0	+/3	–/0	+/3	–/0	–/0	–/0	+/3	–/0	–/0
2	+/2	–/0	–/0	–/0	–/0	–/0	+/3	+/2	–/0	–/0	–/0	–/0	+/3	–/0	–/0
3^a^	+/3	–/0	–/0	–/0	–/0	–/0	+/3	–/0	–/0	–/0	–/0	–/0	–/0	–/0	–/0
4	–/0	–/0	–/0	–/0	–/0	–/0	+/3	+/2	–/0	–/0	+/2	–/0	–/0	–/0	–/0
5	–/0	–/0	–/0	–/0	–/0	–/0	+/3	–/0	–/0	–/0	–/0	–/0	+/3	–/0	–/0
6	+/2	–/0	–/0	–/0	+/2	–/0	+/3	+/3	–/0	–/0	–/0	–/0	+/3	–/0	–/0
7	+/3	+/3	–/0	–/0	–/0	–/0	+/3	–/0	+/3	–/0	+/2	–/0	+/2	–/0	–/0
8	+/3	–/0	–/0	–/0	–/0	–/0	+/3	–/0	–/0	–/0	–/0	–/0	+/3	–/0	–/0
9	+/2	+/3	–/0	–/0	–/0	–/0	+/3	+/3	+/3	+/2	–/0	–/0	+/3	–/0	–/0
10	+/3	+/3	–/0	–/0	+/2	–/0	+/3	+/3	+/3	–/0	+/2	–/0	+/3	–/0	–/0
11^b^	+/2	–/0	–/0	–/0	+/2	–/0	+/3	+/3	–/0	–/0	–/0	–/0	+/3	–/0	–/0
12	+/3	++/2	+/2	–/0	+/2	–/0	+/2	+/3	+/3	+/3	–/0	+/2	+/3	–/0	+/2
13^c^	+/3	++/2	++,+++/3	+/3	+/2	+/3	+++/3	++,+++/3	++/3	++/3	+/2	–/0	+,++/3	–/0	++/3
14	+/3	++/2	+,++/2	–/0	+/2	+/2	++/3	+/3	+/3	+/3	+/2	+/3	+/3	–/0	+/2
15	+/2	+/3	+/3	–/0	+/2	–/0	++/3	+/3	+/3	+/3	+/2	–/0	+/2	–/0	+/2
16	+/2	++/2	+/2	–/0	+/2	–/0	++/3	+/3	+/2	+/2	+/2	–/0	+/3	–/0	+/2
17	+/2	++/3	+/2	–/0	+/2	–/0	++/3	+/3	+/3	+/3	+/3	–/0	+/3	–/0	+/2
18	+/2	++/3	+,++/3	–/0	+/2	–/0	++/3	+,++/3	+/2	+/3	+/3	–/0	+/3	–/0	+/2
19	++/3	++/3	+,++/3	–/0	+/2	–/0	+++/3	++/3	+/3	+/2	+/3	+/2	+/3	–/0	+/2
20	+/2	+/2	+/2	–/0	–/0	–/0	+/3	+/2	+/2	+/2	+/3	–/0	+/2	–/0	–/0
21	+/2	+/3	+/2	–/0	+/3	–/0	+/2	+/3	+,++/3	–/0	+/2	–/0	+/3	–/0	–/0
22	+/2	+/3	+/3	–/0	+/2	–/0	+/2	+/3	+/3	+/3	–/0	–/0	+/2	+/2	+/2
23	+/3	+/3	+/3	–/0	+/2	–/0	+/3	+/2	+/3	+/2	+/2	–/0	+/3	–/0	–/0
24	+,++/3	+++/3	+/3	–/0	+/3	–/0	+,+++/3	+/2	+/3	+/2	+/2	–/0	+/3	–/0	+/2
25^d^	+,++/2	+,+++/3	++/3	+/3	+/3	–/0	++/3	+,+++/3	+/3	+,++/3	+/2	+/3	+,+++/3	+/3	+/2
26	++/3	+++/3	+/3	+/3	+/2	–/0	+,++/3	++/3	+,++/2	+/3	+/2	–/0	+/3	–/0	+/2
27^e^	++/3	+/3	+/2	–/0	+/2	–/0	+/3	+/2	–/0	+/3	+/3	–/0	+,++/3	–/0	–/0
28	–/0	–/0	–/0	–/0	–/0	–/0	–/0	–/0	–/0	–/0	–/0	–/0	–/0	–/0	–/0
29	–/0	–/0	–/0	–/0	–/0	–/0	–/0	–/0	–/0	–/0	–/0	–/0	–/0	–/0	–/0
30	+/3	–/0	–/0	–/0	–/0	–/0	+/2	–/0	–/0	–/0	–/0	–/0	–/0	–/0	–/0

*CD, cluster of differentiation; FoxP3, Forkhead Box P3; HLA, human leukocyte antigen (synonym major histocompatibility complex); IFN, interferon; Ig, immunoglobulin; IL, interleukin; TGF, transforming growth factor; Pax-5, paired box 5; NE, not evaluated. Semiquantitative analysis of immunopositive cells: –, no; +, <10%; ++, 10–50%; +++, 50–90%, and ++++, >90% immunopositive cells. Subjective labeling intensity score of immunopositive cells: 0, 1, 2, and 3*.

a *Multisystemic hyphate mycosis (lung, kidney)*.

b *Hyphate mycosis (lung)*.

c *Suspect Brucella coinfection (primarily CNS lesions)*.

d *Sepsis by Staphylococcus aureus (particularly severe CNS and lung lesions)*.

e *Multisystemic hyphate mycosis (lung, cerebrum)*.

Brain tissue from “control” dolphins had no immunoreactivity to any of the cytokines evaluated (IFNγ, IL4, IL10, TGFβ); however, CeMV-infected striped dolphins and bottlenose dolphins had consistent IFNγ (Figure 10D) and occasional IL4 immunoexpression in CNS tissue sections examined. Overall, there was a more consistent number of IFNγ+ cells in all these cases, but scattered IL4+ cells could be also observed. IFNγ was mainly expressed by MNCs and neuroparenchymal elements. IL4 was expressed by scattered circulating MNCs. Five striped dolphins and one bottlenose dolphin had detectable TGFβ and/or IL10 immunoexpression. The latter involved MNCs in inflamed choroid plexus of fourth ventricle (case 25).

## Discussion

Comparative analyses of LIRs are of paramount relevance as they help unraveling local immunopathogenetic mechanisms of disease processes. While in humans and in given domestic and livestock animal species, as well as in laboratory animals there is a considerable body of knowledge, the immunopathogenetic bases of disease including LIRs in cetaceans remain largely unknown (11). We have herein characterized, by means of a set of lymphocytic, histiocytic, antigen-presenting cell-, cell death-related and cytokine immunomarkers, LIRs among CeMV-infected dolphins from the Western Mediterranean as well as from the Northeast-Central and the Southwestern Atlantic, with special emphasis on their lymphoid, pulmonary, and CNS tissues. There are no previous data on the LIRs in cetaceans infected by CeMV, except for a previous study focused on PBLs in a set of free-ranging bottlenose dolphins with subclinical infection (11). In that study, dolphins with subclinical CeMV infection had elevated lysozyme concentrations, marginally significant increases in monocytic phagocytosis, reduced mitogen-induced T lymphocyte proliferation responses, and marginally significant CD4+ decreases in PBLs compared to seronegative dolphins (11). We will discuss our results tissue by tissue and correlate them with the tissue-specific immunopathology and immunopathogenesis of MeV (6) and CDV infections (5) whenever appropriate. This IHC-based approach was coupled with inferred CeMV disease presentation forms for all animals included in this study (data published elsewhere).

Humoral and cellular immune responses to MeV (6, 19) and CDV (20–23) are crucial for viral clearance and recovery, as well as for the establishment of a “lifelong” protective immunity. However, they are also the pathological basis of measles and distemper morbidity and mortality (5, 9). In both, early immunosuppression is associated with viremia and lysis of lymphocytes and macrophages (6, 24). B-cells, follicular dendritic cells, and T-cells, especially CD4+, and CD8+ cells, are initially targeted by MeV and CDV resulting in generalized lymphoid depletion in lymph nodes and spleen, mucosa-associated lymphoid tissue (MALT), and tonsils (5, 6). Hyperplasia of reticular cells (sinus histiocytosis) in the medullary region of lymph nodes typically accompanies this early phase of infection (24, 25). In this study, we observed consistently decreased numbers of T cells and B cells in Guiana dolphins, striped dolphins, and bottlenose dolphins; however, lymphoid cell depletion appeared to be more severe in Guiana dolphins and in acute/subacute cases from the Canary Islands (cases 13, 14, and 19) and Mediterranean Sea (cases 21, 25, 26, and 27). Additionally, we observed increased sinus histiocytosis (Iba1-, CD68-, and lysozyme-expressing cells) in these dolphins, recapitulating features observed in acute/subacute measles and distemper (5, 9).

CAS3 is a frequently activated death protease, catalyzing the specific cleavage of many key cellular proteins and playing a major role in apoptosis (26). In MeV and CDV infections, upregulation/overexpression of CAS3 is commonly observed in infected lymphocytes and uninfected lymphocytes, suggesting the existence of virus-dependent, and virus-independent mechanisms of apoptosis (27–29). We detected an overall increased CAS3 expression mainly in lymphocytes and histiocytes of cortex and paracortex, but to a lesser extent in histiocytes of subcortical sinuses and medullary cords in lymph nodes. These findings suggest apoptosis is a major cell death mechanism (30) also in the time course of CeMV infection, as it is in measles and distemper (27, 31, 32). Although in these cases viral-induced apoptosis may be the major triggering factor (27, 31, 33), the pathogenetic intricacies of such phenomenon, including viral-independent apoptosis pathways remain undetermined in CeMV infection. Altogether, the above findings concerning lymphocytic and histiocytic disarrangements and generalized immunoexpression of CAS3 in CeMV-infected dolphins provide compelling morphological and IHC evidence of a compromised immune response capacity in these animals.

If the host survives, incipient MeV and CDV infections are generally followed by regeneration of lymphoid organs (5, 6). Repopulation and germinal center formation in lymphoid tissues from persistently infected and convalescent hosts are common in this stage (5, 6). Detection of such “persistently infected” and/or “convalescent” dolphins is complicated; however, we observed similar regenerative findings in several cases from the Canary Islands and the Mediterranean Sea largely involving those animals with chronic systemic and chronic localized “brain only forms” of CeMV infection [supported by IHC and PCR results [data published elsewhere]] (1, 14). These changes were characterized by follicular, paracortical, and medullary cord expansion (reactive hyperplasia), typically encompassing distorted or poorly delineated lymphocytic growths including T cells and B cells accompanied by slightly increased numbers of IgG+ cells. Thus, we detected IHC evidence of a somewhat similar immune response progression in CeMV-infected dolphins compared to MeV- and CDV-infected individuals (5, 6). Nonetheless, further conclusions are limited by the lack of other means of infection's chronology surrogates, including serological profiling. Future studies addressing these limiting factors would allow more accurate comparisons with TMVs. Interestingly, morphologically intact appearing compartments after lymphoid repopulation and virus clearance from lymphoid tissues does not necessarily result in complete functional regeneration of the immune response (22, 34, 35). This may also apply to CeMV infection, so that apparently “normal” lymphocytic density and distribution patterns in lymphoid districts from CeMV-infected cetaceans may not necessarily be associated with appropriate immunological fitness. In chronic measles and distemper, often this multicentric repopulation is accompanied by CD4- and CD8-dominated inflammatory responses in the CNS (22, 36, 37). This remains to be evaluated in cetaceans.

Mammals differ in their expression of MHCII molecules (38). In this study, we observed MHCII expression was largely confined to MNCs consistent with B-lymphocytes, dendritic cells, and macrophages (especially sinus histiocytes) which are considered professional antigen-presenting cells (38). Also, there were small numbers of MHCII-expressing MNCs in the paracortex and subcortical medullary cords, diminishing centripetally. This distribution pattern is consistent with reports in other species, including rodents (38); however, more accurate inferences may be obtained by double immunolabeling analyses. We suspect certain T-cell lymphocytes could also express MHCII in CeMV-infected animals; however, greater T cell numbers, including resting ones, as reported in pigs, dogs, cats, mink, and horses (38), were not a feature in CeMV-infected or “control” animals. No significant alterations in MHCII immunoexpression were observed between infected and control animals on the basis of semiquantitative comparative analysis.

Comparatively, fewer studies have addressed in detail the immune disarrangements in the spleen of MeV and CDV cases (5, 6). Typically, there is a generalized depletion of T and B cell compartments in the spleen (5, 22). In our study, most findings observed in lymph nodes were paralleled to some extent by those detected in the spleen, with divergences dependent upon T-cell and B-cell topographic locations. There was an overall decreased number of T cells and B cells in PALS and follicles, respectively, along with an increased CAS3 immunoreactivity and increased number of histiocytes. These findings are in agreement with observations in MeV and CDV infections (5, 6, 22). IgG-expressing cells were slightly increased, as reported in measles (39).

There is relatively limited knowledge on cytokine networks and interplay in LIR in TMV infections, especially concerning IHC investigations. In cetaceans, there is a complete lack of information on “*in situ*” cytokine expression. Th1 responses evoke cell-mediated immunity and phagocyte-dependent inflammation. Th2 cells evoke strong antibody responses and eosinophil accumulation, but inhibit several functions of phagocytic cells (phagocyte-independent inflammation). Furthermore, Th1/Th2 balance can be evaluated by the *ratio* of their polarizing cytokines (i.e., IFNγ/IL4), and animals with imbalanced Th1/Th2 response may be more susceptible to certain kinds of infections. Since cytokine imbalance is implicated in the pathogenesis and outcomes of MeV and CDV infections, we aimed at evaluating, for the first time, Th1 and Th2 *in situ* cytokine immunoexpression and potential associations with CeMV-AD. We employed a set of commercially available non-cetacean-specific but cross-reactive proinflammatory cytokines to evaluate Th1 cells (IFNγ-secreting) and Th2 cells (IL4-, IL10-secreting) (13). In distemper, a lack of detectable cytokine expression in peripheral blood leukocytes is associated with a high viral load and viremia, indicating that an overwhelming virus infection may suppress cytokine production in lymphoid cells (40, 41). Plasma IFNγ levels (consistent with a predominant Th1 immune response) are increased during the acute phase of measles, whereas, a subsequent Th2 response promotes the development of protective MeV-specific antibodies and is characterized by high concentrations of IL4, IL10, IL13, and IL17 (9). This shift may promote B cell maturation and contribute to the continued production of antibody-secreting cells (9). In the present study, no IHC-based differences were detected regarding cytokine immunoreactivity in lymph nodes and spleen between infected and uninfected dolphins. Severe cytokine “storms” are often ascribed as to the cause of multiorgan dysfunction and death in infectious diseases. IHC analysis may not be as sensitive and specific as PCR-based mRNA transcript quantification; therefore, ongoing studies are aimed at quantifying cytokines by molecular techniques to better address this issue.

Few studies have evaluated the LIR to MeV infection in lung (42) and presumably no information is available on CDV-associated LIR (5). MeV-infected children may display severe depletion of CD4+, CD20+, CD68+, NK+, and S100+ cells in alveoli and BALT without depletion of CD8+ T-cells (42). Also, there is prominent apoptosis of dendritic, CD4+ and NK cells (42). In our study, comparisons made with “control” dolphins—all of which were regarded as calves—revealed mild to moderate increase in T cells and B cells and histiocytes in lung of CeMV-infected dolphins, which also contrasts with observation in children with measles (42). Further studies involving “age-matched control” dolphins, an extremely difficult setting when dealing with free-ranging cetaceans, may help elucidate potential age-related bias in CeMV-LIRs, including lung tissue. Furthermore, there were increased CAS3+ cells in CeMV-infected dolphins mainly involving inflammatory MNC populations and epithelial cells (27). The number and intensity of CAS3-expressing cells was greater in parasitic inflammatory foci. This novel finding also adds to the limited available knowledge on the immunopathogenesis of verminous pneumonia in dolphins (43). Cases 3, 11, and 27 had concomitant pulmonary hyphate mycosis, which accounted for increased numbers of lymphocytes, macrophages, and exudating neutrophils and necrosis, primarily associated with fungal elements. Fungal coinfections are common and exacerbate pneumonia in measles (44), distemper (45), and CeMV infections (46–48). Case 25 presented a consistently expanded BALT and alveolar septa harboring abundant T cells and B cells. To the authors' knowledge, this inflammatory pattern is unusual in dolphins, yet it recapitulates features of *Mycoplasmataceae* infection (“cuffing pneumonia”) in other *Cetartiodactyla* members. The etiopathogenesis of this finding remains unknown. Furthermore, we detected increased expression of MHCII chiefly involving circulating MNCs, including presumed PIVMs, and infiltrating inflammatory MNCs (lymphocytes and histiocytes) and exocytosing/exudating MNCs. This was more prominent in parasitic LIRs. Putative PIVMs appeared to consistently express Iba1, lysozyme and CD68; these results broaden the repertoire of immunomarkers for PIVMs in cetacean species (49). IgG+ lymphocytes were mainly around bronchioles/bronchi (in BALT) and scattered in the interstitium, especially in dolphins with concomitant verminous pneumonia. No evident differences were detected for CD57- and FoxP3-expressing cells in lymphoid organs between infected and uninfected cetaceans.

Scarce studies on measles lung LIR indicated depletion of IL-10+ and IL-12+ cells in infected children; however, there was a greater number of IL-1+, IFN+, and IL-4+ cells (42). Recently, IFNγ-secreting cells were shown to be more abundant early and IL-17+ cells late in lung of rhesus macaques (*Macaca mulatta*) experimentally infected with wild type MeV. Both CD4+ and CD8+ T cells were sources of IFNγ (19). In the present study, we detected some “basal” immunoreactivity for IFNγ, IL4, IL10, and TGFβ in lung tissue of “control” dolphins and even though these cytokines appeared expressed by a slightly greater number of MNCs in lung of infected dolphins we did not find evident differences based on the proposed IHC semiquantitative approach. To better address potential cytokine differences in lung between CeMV-infected and uninfected dolphins we are currently focusing on quantitative molecular cytokine analysis.

The three main MeV-CNS complications include: acute disseminated encephalomyelitis, measles inclusion body encephalitis, and subacute sclerosing panencephalitis (SSPE). CDV-CNS disease may show distinctive manifestations: acute fulminant encephalopathy and encephalitis, post-vaccinal encephalitis, old dog encephalitis, inclusion body polioencephalitis, and demyelinating leukoencephalitis (CDV-DL) (5, 50). Their etiopathogenesis is known to vary and except for SSPE and CDV-DL, their LIR remain largely undetermined (51–53). In this study, we observed varying degrees of CNS inflammation in CeMV-infected dolphins, predominantly in striped dolphins and bottlenose dolphins with DMV. Overall, the LIR in these cases was dominated by (in decreasing order) by T cells, B cells, and histiocytes accompanied by scattered IgG**+** plasma cells, regardless of the CeMV-associated presentation. These findings, particularly concerning lymphocytic LIR in CeMV-infected dolphins resemble CNS lymphocytic LIR in measles (53) and distemper (54); however, an in-depth analysis of CD4+ and CD8+ lymphocytes (both subpopulations representing CD3+ cell subtypes) is hampered by the lack of reliable CD4 and CD8 markers applicable in cetacean FFPE tissues (13). Divergences of CeMV-CNS LIR were evident in cases 13 and 25. The former was a suspect case of CeMV and *Brucella* coinfection. In this case, a greater number of histiocytes, multifocally characterized by a granulomatous phenotype and B cells were observed. Case 25 had a confirmed septicemia by *Staphylococcus aureus*, with major CNS involvement (16). Immunophenotypic divergences in the latter included greater number of histiocytes, along with the presence of a suppurative exudate compared to the other herein investigated genuine cases of CeMV infection with CNS involvement. CD57- and FoxP3-expressing cells were very rarely detected in CNS tissue sections.

We observed consistently increased numbers of CAS3+ in CNS tissue, mainly involving lymphocytes and histiocytes circulating and infiltrating the meninges, expanding the VRS and infiltrating the neuroparenchyma. Occasionally, neurons and neuroglia expressed CAS3 in areas of marked neurodegeneration. These findings are in agreement with previous observations in MeV- and CDV-neurologic disease (55, 56). MHCII was only expressed in CeMV-infected dolphins, thereby involving a consistent fraction of the aforementioned inflammatory cells and occasionally vascular cells. Neuroglial cells, namely microglia and astrocytes, rarely expressed MHCII. MHCI, a major viral antigen-presenting molecule, awaits development of reliable antibodies for use in FFPE cetacean tissues (13). Overall, these findings are in agreement with previous reports of MeV- and CDV-associated neurological disease (8, 39, 51, 53, 57).

Previous studies suggested there is a cytokine imbalance in SSPE. INFγ and TNFα are overexpressed in endothelial and glial cells from SSPE-affected patients (51). Furthermore, in CDV-DL, there is IHC evidence of increased pro-inflammatory cytokines such as IL1, IL6, IL8, IL12, and TNF in early stages, whereas, IL1, IL6, and IL12 would predominate in advanced diseases stages (58). By contrast, the expression of anti-inflammatory cytokines, e.g., IL-10 and TGF-β appears to be restricted to animals with inactive or chronic disease stages (59). Interestingly, the cerebrospinal fluid of naturally infected dogs may contain detectable levels of TNFα and IL-6 mRNA as well as of IL10 and TGFβ RNA transcripts simultaneously, so that the staging of the disease becomes troublesome (5). In the present study, CeMV-infected striped dolphins and bottlenose dolphins had consistent IFNγ and occasional IL4 immunoexpression in CNS tissue sections examined. Overall, there was greater number of IFNγ+ cells in all these cases, suggesting a Th1-CNS polarization at the time of death, regardless acute or chronic CeMV-AD presentation. By contrast, brain tissue from “control” dolphins appeared immunologically quiescent (60). IFNγ is pivotal in the CNS-MeV infection (61). Deficient CNS-MeV IFNγ responses render individuals highly susceptible (62). The IFNγ immunoreactivity observed in the present study suggests IFNγ plays a role in the infection's neuropathogenesis also in CeMV-infected dolphins. The detection of occasional simultaneous IL4+ cells suggests Th1/Th2 cytokine interplay during the course of CeMV. Finer quantitative methodologies and *in vitro* analyses may allow better assessment of the roles of these two “mutually inhibitory” cytokines in CNS-CeMV infection. The participation of IL10+ and TGFβ+ cells, detected in much smaller numbers in CNS tissue sections, remains unclear. Further studies are necessary to understand the neuroimmunopathogenesis of CeMV infections, with special emphasis on cytokine networks.

To the best of our knowledge, this study represents the first attempt to characterize and compare the LIR in cetaceans infected with CeMV other than in PBLs (11). It would be appropriate to comment on various limiting factors of the present study, some inherent to dealing with carcasses of free-ranging cetaceans and some inherent to laboratory diagnostics. Although we prioritized fresh carcasses, some of the tissues showed mild *post-mortem* autolysis/decomposition *phenomena* that could have negatively interfered with IHC reactions. Additionally, although we used pAbs standardized for their use in cetacean species and we based our judgment(s) of suitable cross-reactivities upon cellular morphologic features and cellular immunostaining patterns similar to human and mouse and other cetacean control tissues (13), future studies involving cetacean-specific antibodies should revisit these findings. A relevant factor in this study concerned the “control” group. In this respect, it is extremely difficult to find “perfectly healthy” or “gold-standard” control animals in natural settings, with special reference to free-ranging cetaceans; however, the application of strict inclusion criteria (targeting at least one individual for each species) retrieved three calves. Based on histologic analysis and IHC results, these animals had developed lymphoid systems and did not show overt variations in comparison to a previous report including a female juvenile and two male calves (13). Thus, no evident age-related bias was readily apparent in these animals and comparisons with CeMV-infected dolphins were deemed to be appropriate.

These results indicate a complex interplay between lymphocytic, histiocytic, antigen-presenting cell-, cell death-related, and cytokine elements in LIRs to DMV and GD-CeMV infections in striped dolphins, bottlenose dolphins, and Guiana dolphins, respectively. We detected consistent CeMV-associated inflammatory response patterns with some similarities and few differences between DMV-infected striped and bottlenose dolphins, and GDCeMV-infected Guiana dolphins. These are summarized as follows. In the lymphoid system (lymph nodes, spleen), (a) there was multicentric lymphoid depletion, characterized by reduced numbers of T cells and B cells in all three species infected by CeMV; however, lymphoid depletion phenomena were more pronounced in Guiana dolphins infected with GDCeMV; (b) striped dolphins and bottlenose dolphins, infected with DMV, often had hyperplastic (regenerative) phenomena involving the aforementioned cell populations, particularly chronically infected animals; (c) there was generalized increased expression of caspase 3 in all three species; and (d) no differences were detected regarding cytokine immunoreactivity. In the lung, (a) there was a mild to moderate increase in T cells, B cells, and histiocytes in all three species; and (b) no differences were detected regarding cytokine immunoreactivity. Concerning the CNS: (a) inflammation was a consistent feature in DMV-infected striped and bottlenose dolphins in contrast to Guiana dolphins infected by GDCeMV; (b) inflammation was dominated by T cells and B cells, accompanied by fewer Iba1+, CD68+, and lysozyme+ histiocytes; (c) there was increased expression of caspase 3; and (d) no differences were detected regarding cytokine immunoreactivity except for IFNγ in the CNS of infected dolphins of all three species.

In conclusion, these novel results delineate the local immunophenotypic response during CeMV infection in three highly susceptible delphinid species. They also suggest a complex interplay between viral and host's immune factors, thereby contributing to gain valuable insights into similarities and differences of CeMV infection's immunopathogenesis in relation to body tissues, CeMV strains and cetacean hosts. Finally, the herein presented IHC investigation results may help elucidating the immunopathogenetic bases, including the kinetics of LIRs in other infectious and non-infectious disease processes in cetaceans, with major applications in ecotoxicological pathology.

## Data Availability

All data for this study are included in the manuscript and/or the supplementary files.

## Author Contributions

JD-D, KG, and JC-D contributed conception and design of the study. JD-D, KG, ES, SS, ÓQ-C, MA, AF, ES-N, JI, RC, JL-B, LF, CC, SM, LD, GDF, GDG, and JC-D contributed to organization of the databases and/or collected samples for histopathological, immunohistochemical, and molecular analyses. JD-D, KG, RR, IR, CK, NF, and BC contributed to immunohistochemical analyses and laboratorial resources. JD-D, KG, ES, CC, LD, and GDF conducted molecular analyses. JD-D wrote the first draft of the manuscript. All authors contributed to manuscript revision, read and approved the submitted version.

### Conflict of Interest Statement

The authors declare that the research was conducted in the absence of any commercial or financial relationships that could be construed as a potential conflict of interest.
