# Supplementary material for: Comparative Immunopathology of Cetacean morbillivirus Infection in Free-Ranging Dolphins From Western Mediterranean, Northeast-Central, and Southwestern Atlantic
Source: Front Immunol. 2019 Mar 18;10:485. doi: 10.3389/fimmu.2019.00485 (PMC6431672; doi:10.3389/fimmu.2019.00485)
Supplement: Supplementary file 1 [file Table_1.DOCX]

**Table S1.** Gross and microscopic pathologic findings in a striped dolphin (*Stenella coeruleoalba*), bottlenose dolphin (*Tursiops truncatus*) and a Guiana dolphin (*Sotalia guianensis*) used as ‘control’ in the present study.

| **No** | **Gross** | **Microscopic** |
| --- | --- | --- |
| 28 | **Eye**: Multifocal periocular hemorrhage in the right eye. **Skin**: Multifocal irregular erosions and ulcers in right lateral and ventral aspect of head and thorax; Two deep circular ulcers in the ventral aspect of the head and thorax; Bilateral hematoma in the subcutis of pectoral fins; Bilateral hemorrhage in the head and neck. **Musculoskeletal system**: Multifocal acute hemorrhage in ventral and lateral cephalic musculature. **Esophagus**: Multifocal erosive-ulcerative esophagitis. **Keratinized and glandular gastric chambers**: Small amount of digested ingesta (squid beaks, lenses and otoliths) and multifocal hemorrhage. **Pyloric chamber**: Dilated, with abundant dark fluid and multifocal hemorrhage. **Lung**: Marked, bilateral edema and partial atelectasia. **Prescapular lymph node**: Mild lymphadenomegaly; Spleen: Focal subcapsular hemorrhage. **Encephalon and cervical spinal cord**: Focally extensive, suffusive subdural hemorrhage. | **Skin:** Mild, focal, chronic ulcerative dermatitis and epidermal hyperplasia; Mild, multifocal, chronic lymphohistiocytic dermatitis and panniculitis. **Skeletal muscle:** Mild, multifocal myocyte atrophy; Multifocal interstitial edema; Scattered neutrophilic and lymphocytic interstitial infiltrates; Scattered hemorrhage in prescapular fascia. **Esophagus**: Multifocal ulcerative esophagitis. **Glandular stomach**: Erosive and hemorrhagic gastritis. **Lung**: Marked, diffuse alveolar edema with alveolar histiocytosis, keratin squames and patchy atelectasia; Abundant mineralizations. **Mesenteric lymph node:** Minimal, multifocal pyogranulomatous lymphadenitis, mild sinus histiocytosis and scattered hemosiderosis. **Spleen**: Subcapsular hemorrhage; Extramedullary hematopoiesis. **Kidney**: Congestion and interrenicular edema; Rare tubular mineralization. **Pancreas**, **mediastinal and prescapular lymph node**, **Keratinized stomach**, **Colon**, **Cerebellum**, **Cerebrum**: NSLO. |
| 29 | **Skin:** Multifocal irregular erosions on the lateral and ventral aspect of head; multifocal subcutaneous petechiae and edema. **Trachea and lung**: Marked bilateral edema. **Thorax and pleura:** Multifocal petechiae throughout the pleurae and epicardium. **Peritoneum:** Petechiae. **Mesenteric lymph nodes**: Moderate lymphadenomegaly. **Spleen and liver**: Moderate diffuse congestion. **Urinary bladder**: Mucosal petechiae. **Keratinized and glandular gastric chambers:** Multifocal erosion and ulcers (keratinized stomach), and gastritis (glandular stomach). **Intestine:** Diffuse moderate catharral enteritis. **Encephalon:** Meningeal edema and congestion. | **Heart**: Mild, multifocal cardiomyocyte vacuolation; Focal, acute epicardial hemorrhage. **Keratinized stomach**: Moderate, focal, subacute ulcerative gastritis. **Glandular stomach**: Minimal, focal crypt dilation and cryptitis. **Spleen**: Moderate, diffuse extramedullary hematopoiesis. **Mediastinal** **lymph node**: Mild, diffuse, lymphoid depletion with sinus edema, fibrin and hemorrhage and mild erythrophagocytosis. **Thyroid**: Mild congestion. **Skeletal muscle**: Mild, multifocal, acute myonecrosis with satellitosis/phagocytosis. **Umbilical artery**: incomplete thrombosis with hemorrhage. **Adrenal gland**: Mild, multifocal, acute cortical hemorrhage with single cell degeneration and loss. **Lung**: Moderate to marked, diffuse pulmonary atelectasia, edema and mild aspirated squames, minimal meconium with bronchochonstriction and minimal to mild acute interstitial pneumonia and alveolar histiocytosis. **Trachea**: Mild, multifocal, acute neutrophilic tracheitis with hemorrhage and epithelial necrosis and loss and vasculitis. **Small intestine:** scattered crypt ectasia and cryptitis, some containing meconium. **Colon**, **Pancreas, Urinary bladder, Urethra, Ureters, Laryngeal tonsil, Pylorus**: NSLO. |
| 30 | **Skin**: Diffuse icterus. **Lung**: Bilateral pulmonary edema with multifocal hemorrhage. **Liver**: Diffuse lipidosis. | **Lung**: Marked, diffuse alveolar edema with scattered acute hemorrhage and rare squames. **Spleen**: Diffuse congestion with scattered acute hemorrhage; Mild extramedullary hematopoiesis. **Small intestine**: Focal luminal hemorrhage. **Lymph node**: Diffuse congestion; Multifocal sinus vascularization; Mild sinus edema and histiocytosis. **Liver**, **Esophagus**, **Trachea**: Congestion. **Kidney**: Moderate congestion. **Skin**: Mild, diffuse, chronic hyperkeratosis. **Skeletal muscle**, **Heart**, **Lymph node**, **Cerebrum**, **Stomach**, **Aorta**: NSLO. |

NSLO, no significant lesions observed.
