# Supplementary figures and images for: Comparative Immunopathology of Cetacean morbillivirus Infection in Free-Ranging Dolphins From Western Mediterranean, Northeast-Central, and Southwestern Atlantic
Source: Front Immunol. 2019 Mar 18;10:485. doi: 10.3389/fimmu.2019.00485 (PMC6431672; doi:10.3389/fimmu.2019.00485)

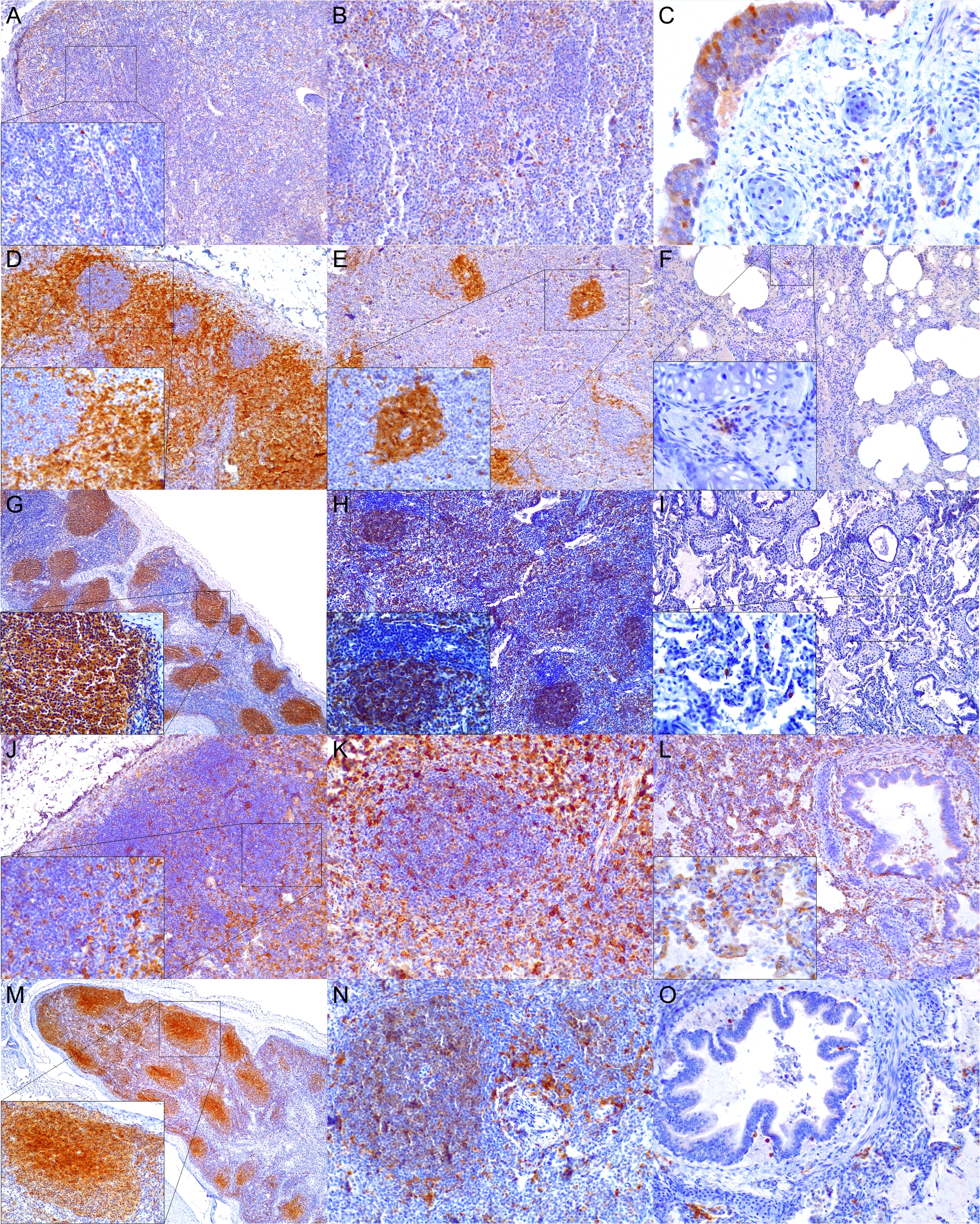

Supplement: Supplementary file 2 [file Image_1.JPEG]
